# Age-Dependent Modulation of Synaptic Plasticity and Insulin Mimetic Effect of Lipoic Acid on a Mouse Model of Alzheimer’s Disease

**DOI:** 10.1371/journal.pone.0069830

**Published:** 2013-07-17

**Authors:** Harsh Sancheti, Garnik Akopian, Fei Yin, Roberta D. Brinton, John P. Walsh, Enrique Cadenas

**Affiliations:** 1 Department of Pharmacology and Pharmaceutical Sciences, School of Pharmacy, University of Southern California, Los Angeles, California, United States of America; 2 Davis School of Gerontology and Program in Neuroscience, University of Southern California, Los Angeles, California, United States of America; University of Ulster, United Kingdom

## Abstract

Alzheimer’s disease is a progressive neurodegenerative disease that entails impairments of memory, thinking and behavior and culminates into brain atrophy. Impaired glucose uptake (accumulating into energy deficits) and synaptic plasticity have been shown to be affected in the early stages of Alzheimer’s disease. This study examines the ability of lipoic acid to increase brain glucose uptake and lead to improvements in synaptic plasticity on a triple transgenic mouse model of Alzheimer’s disease (3xTg-AD) that shows progression of pathology as a function of age; two age groups: 6 months (young) and 12 months (old) were used in this study. 3xTg-AD mice fed 0.23% w/v lipoic acid in drinking water for 4 weeks showed an insulin mimetic effect that consisted of increased brain glucose uptake, activation of the insulin receptor substrate and of the PI3K/Akt signaling pathway. Lipoic acid supplementation led to important changes in synaptic function as shown by increased input/output (I/O) and long term potentiation (LTP) (measured by electrophysiology). Lipoic acid was more effective in stimulating an insulin-like effect and reversing the impaired synaptic plasticity in the old mice, wherein the impairment of insulin signaling and synaptic plasticity was more pronounced than those in young mice.

## Introduction

Alzheimer’s disease is a neurodegenerative disorder characterized by brain accumulation of senile amyloid-β plaques and hyperphosphorylated tau (neurofibrillary tangles) in the medial temporal lobe and cortical areas of the brain [1]. Alzheimer’s disease is the most prevalent neurodegenerative disease among the aging population [1] and a leading cause of dementia with progressive memory deficits, cognitive impairments, and personality changes. Support for an early mitochondrial dysfunction that precedes the histopathological hallmarks described above in Alzheimer’s disease continues to increase [2,3,4,5]. Perturbations of mitochondrial function in terms of altered morphology, compromised electron transfer complexes, and tricarboxylic acid cycle deficiencies have been long identified in post-mortem tissues of Alzheimer’s patients [6,7].

Multiple levels of analyses indicate a dysfunction of glucose metabolism and mitochondrial bioenergetics as antecedents to the development of Alzheimer’s pathology [8,9,10,11]. A decline in brain glucose uptake (and metabolism) can appear decades prior to the onset of histopathological changes inherent in Alzheimer’s disease: several independent clinical studies showed that decreased brain glucose uptake is a common condition in patients with Alzheimer’s disease and mild cognitive impairment (MCI) [12,13]. A prominent decrease of glucose uptake in several types of dementias, i.e., MCI, dementia Alzheimer’s type (DAT), frontotemporal dementia, and dementia with Lewy bodies has been demonstrated in multicenter clinical studies of 548 patients [14].

Human brain has the highest consumption of glucose with respect to its size (60% of body’s resting state glucose) and the energy generated from glucose metabolism is essential to support synaptic transmission [15]; as a corollary, synaptic transmission is susceptible to the bioenergetic deficits associated with the progress of Alzheimer’s disease [16,17]. The insulin-stimulated brain glucose uptake knits and links brain insulin to synaptic transmission. Insulin has been shown to influence synaptic transmission by modulating the cell membrane expression of NMDA (N-methyl-D-aspartic acid) receptors and, thereby affect long-term potentiation (LTP) [18]. Hence, insulin resistance (as it occurs in metabolic syndrome) can modulate cognition and deteriorate other brain functions [19]. The disruption of brain glucose uptake and metabolism following insulin resistance is linked to Alzheimer’s disease and ‘treatment of brain insulin resistance’ is being widely considered as a therapeutic approach in Alzheimer’s disease [20]. Alzheimer’s disease is widely associated with synaptic failure, resulting in the loss of declarative and nondeclarative memory and is associated with massive brain atrophy over a period of time [21]. Long-term potentiation (LTP) is considered as a major cellular mechanism underlying learning and memory [22]. The classical pathology associated with Alzheimer’s disease i.e., β-amyloid oligomers, have been shown to impair synaptic plasticity by inhibiting LTP and enhancing long term depression (LTD) [23]. Thus, impaired insulin signaling and subsequent decrease in brain glucose uptake (leading to disturbances in bioenergetics) and compromised synaptic plasticity (leading to disturbances in synaptic transmission) are major deficiencies associated with Alzheimer’s disease.

Lipoic acid (1,2-dithiolane-3-pentanoic acid) was reported to increase glucose uptake in L6 muscle cells and 3T3-L1 adipocytes [24,25], induce the redistribution of GLUT4 to the plasma membrane in 3T3-L1 adipocytes [25], and increase insulin sensitivity in diabetic patients [26]. R-α-lipoic acid -naturally occurring form of the cyclic disulfide- is involved in the regulation of cellular energy (as catalytic cofactor of mitochondrial α-ketoacid dehydrogenases), of transcriptional processes (such as activation of Nrf2 and phase II enzymes), and of kinases and phosphatases involved in signal transduction pathways [27]. These numerous effects of R-α-lipoic acid can be mechanistically viewed in terms of thiol/disulfide exchange reactions that modulate the redox and energy status of the environment; hence, lipoic acid-driven thiol/disulfide exchange reactions appear critical for the modulation of proteins involved in cell signaling and transcriptional pathways [28]. Exogenous lipoic acid equilibrates among different intracellular and extracellular compartments but cannot substitute for covalently bound lipoic acid (as the cofactor of mitochondrial complexes such as pyruvate dehydrogenase and α-ketoglutarate dehydrogenase).

This study is aimed at establishing the effects of lipoic acid, on glucose uptake, insulin signaling through the PI3K/Akt pathway, and synaptic plasticity on a triple transgenic mouse model of Alzheimer’s disease (3xTg-AD). This transgenic model harbors PS1(M146V), APP(Swe), and tau(P301L) transgenes, shows progressive development of both plaques and tangles with increasing age in a region specific manner: ~6 month-old 3xTg-AD mice show diffuse amyloid plaques in different regions but tangle pathology is established at ~12 months [29]. The experimental model consisted of 3xTg-AD mice of these two ages with or without 4 weeks of lipoic acid supplementation in the drinking water. It is hypothesized that an insulin-like effect of lipoic acid could overcome the decreased brain glucose uptake, restore the PI3K/Akt signaling to stimulate the cellular bioenergetics, and reverse the impaired synaptic plasticity in the 3xTg-AD model of Alzheimer’s disease.

## Materials and Methods

### Animal Treatments and Ethics

All rodent experiments were performed following National Institutes of Health guidelines on use of laboratory animals and an approved protocol (protocol number: 11211) by the University of Southern California Institutional Animal Care and Use Committee. The presented study has been approved by the University of Southern California Institutional Animal Care and Use Committee (Ethics Committee).

### Mice colonies and lipoic acid feeding

Colonies of 3xTg-AD and nonTg mouse strain (C57BL6/129S; gift from Dr. Frank Laferla, University of California, Irvine) were bred and maintained at the University of Southern California. Mice were housed on 12-h light/dark cycles and provided *ad libitum* access to food and water. 6- and 12 month-old mice were used for experiments. 3xTg-AD and nonTg mice were either fed with water containing 0.23% R sodium lipoic acid (gift from Geronova Research, Inc.) or normal water for 4 weeks. Thus, at the time of sacrifice, the mice were ~7 (young mice) or ~13 months (old mice). The terms “young mice” and “old mice” are only for representing the data with simplicity. In regards to the nonTg mice, 13 months may not be technically considered as an old age, however, the 3xTg-AD mice show substantial pathology at this age and thus we used age matched nonTg mice at 13 months terming them as old mice. NonTg and 3xTg-AD mice were used to assess the effects of lipoic acid on glucose uptake, the PI3K pathway of insulin signaling, and synaptic plasticity.

### Brain glucose uptake

We employed positron emission tomography utilizing radiotracer fluoro-2-deoxy-2-[^18^F]-fluoro-D-glucose (FDG-PET) in a clinical setting to measure the brain glucose uptake. Utilizing microPET scanning after 40 min post-injection of [^18^F]-FDG-PET as a tracer, the brain glucose uptake was determined by standard uptake value (SUV). SUV represents the standardized uptake value taking into consideration the ratio of the actual radioactivity concentration found in brain at a specific time point and the concentration of radioactivity, assuming an even distribution of the injected radioactivity across the whole body. Briefly, mice were fasted overnight and then sedated using 2% isoflurane by inhalation and were administered the radiotracer 2-deoxy-2-(^18^F) fluoro-D-glucose intravenously. Mice were placed on the scanner bed with a warming bed to maintain body temperature and underwent scanning using a Siemens MicroPET R4 PET scanner with a 19 cm (transaxial) by 7.6 cm (axial) field of view and an absolute sensitivity of 4% with a spatial resolution of ~1.3 mm at the center of view for a duration no longer than 90 minutes. Blood for glucose baseline measurements was collected before the administration of the tracer and measured to ensure that abnormalities in glucose metabolism during [^18^F] FDG-PET imaging are not due to huge differences in starting blood glucose levels but the intrinsic activity of the brain. Additionally, the animals underwent CT scanning with intravenous contrast material. This provided (~1 mm) information of brain structure. Structural imaging using CT scanning allowed the analysis of functional ([^18^F] FDG-PET) and anatomical data. PET data were all reconstructed using the 2D-OSEM algorithm supplied by microPET manager (Siemens Medical Solutions USA, Inc., Knoxville, TN) into 128×128×63 images with 0.084 mm × 0.084 mm × 1.21 mm resolution. CT scans were acquired in two bed positions using the following settings: 80 kVp, 500 µA, 100 ms/180 steps covering 360 degrees and reconstructed into 768×768×923 images with 0.105 mm isotropic resolution. PET and CT images were co-registered using rigid transformations as both scans were performed using warmed multi-modality imaging chambers. Region of interest were drawn to calculate SUV.

### Blood glucose levels

Briefly, mice were fasted overnight and then sedated using 2% isoflurane by inhalation. The mouse tail was warmed a bit using a lamp or a heating pad. The tail vein was located and a small puncture with a 25mm gauge needle was made. The drops of blood that oozed out were tested for the blood glucose levels using a glucose meter and strips (Abbott, Inc.) as per manufacturers supplied instructions. Blood concentrations were considered basal if they were below 45mg/dl after fasting overnight. Glucose standards were used regularly to ensure the accuracy of the glucose meter.

### Brain homogenate preparation

Mice at ~7 and ~13months, after 4 weeks of lipoic acid feeding in drinking water, and age matched control animals fed normal water were used for the experiments. Age matched groups (3xTg-AD and nonTg ± lipoic acid) were fasted overnight to ensure basal glucose levels. Mice were sacrificed by decapitation and the brains were quickly excised on ice. Whole brain was further washed with isolation buffer, and cut into small pieces and finally homogenized using a loose Teflon homogenizer. The buffer consisted of 250 mM sucrose, 20 mM HEPES, 1 mM EDTA, 1 mM EGTA, 1.0% (w/v) BSA, and 25 µl/100 ml protease inhibitor mixture (Sigma Aldrich, MO, USA; Catalogue #8340) at pH 7.4.

### Crude plasma membrane preparation

Brain homogenate was prepared as explained above. Further, the homogenate was spun at 1000 x g for 15 min to remove pelleted nuclear fraction. The nuclear fraction was then discarded and the remaining supernatant was spun at 9000 x g for 30minutes to yield crude cytosol supernatant and a pellet of crude membrane. The pellet was re-suspended in HEPES-Lysis buffer (50 mM HEPES pH 7.4, 2 mM EDTA, protease/phosphatase inhibitors) and spun at 30,000 x g for 30min to yield membrane fraction as a pellet. The pellet was further re-solubilized in 2% CHAPS and stored at -80° C until used.

### Western blotting

Brain homogenates or membrane preparations were quantified by using BCA protein assay kit (Thermo Scientific, IL). The samples were diluted using 2% CHAPS to equalize the protein concentrations. 25% (of the total sample volume) lane marker non-reducing sample buffer (Thermo Scientific, Inc) was added prior to denaturing the proteins by boiling the samples at 95° C for 10 min. Equal amounts of brain homogenate or membrane proteins (20 µg/well) were loaded in each well of a 10% SDS-PAGE gel, electrophoresed with a Tris/glycine running buffer, and transferred to a 0.45 µm pore size polyvinylidene difluoride (PVDF) membrane and immunoblotted with the appropriate primary antibody. Primary antibody was incubated overnight, followed by washing and probing with the appropriate HRP-conjugated anti-rabbit secondary antibody or HRP-conjugated anti-mouse secondary antibody (Vector Laboratories, Burlingame, CA). The immunoreactive bands were visualized by Pierce SuperSignal Chemiluminescent Substrates or SuperSignal West Pico Chemiluminescent Substrate (Thermo Scientific, IL) and captured by Molecular Imager ChemiDoc XRS System (Bio-Rad, Hercules, CA). All band intensities were quantified using Un-Scan-it software (Silk Scientific, UT).

### Metabolic Flux Analysis: XF-Extraflux Analyzer

Primary cortical neurons from day 14 (E14) embryos of non-Tg mice were cultured on Seahorse XF-24 plates at a density of 75,000 cells/well. Neurons were grown in Neurobasal Medium +B27 supplement for 7 days before experiment. 18 hours before assay, lipoic acid (20 µM) and/or LY294002 (50 µM) were added to medium. On the day of metabolic flux analysis, cells were changed to unbuffered DMEM (DMEM base medium supplemented with 25 mM glucose, 2 mM sodium pyruvate, 31 mM NaCl, 2 mM GlutaMax, pH 7.4) and incubated at 37° C in a non-CO_2_ incubator for 1 h. All medium and injection reagents were adjusted to pH 7.4 on the day of assay. Baseline measurements of oxygen consumption rate (OCR, measured by oxygen concentration change) and extracellular acidification rate (ECAR, measured by pH change) were taken before sequential injection of treatments/inhibitors: oligomycin (ATP synthase inhibitor, 4 µM), FCCP (mitochondrial respiration uncoupler, 1 µM), and rotenone (Complex I inhibitor, 1 µM). After the assays, plates were saved and protein readings were measured for each well to confirm equal cell number/well.

### Long-Term Potentiation and I/O Curves

Preparation of hippocampal slices: Each animal was decapitated after deep isoflurane anesthesia and the brain was rapidly removed and immersed in sucrose-modified artificial cerebrospinal fluid (ACSF) containing (in mM): 105 sucrose; 62 NaCl, 3 KCl, 4 MgCl_2_, 1.25 NaH_2_PO_4_, 26 NaHCO_3_, 10 glucose. After 3-5 min of cooling, the brain was cut to contain the hippocampus and coronal 350 µm thick hippocampal slices with surrounding cortical tissue using a vibratome (Series 1000, St Louis, MO). Sections were then transferred to an incubation chamber, where they remained submerged in oxygenated artificial cerebrospinal fluid (aCSF), which consisted of (in mM), 124 NaCl, 3 KCl, 1.25 NaH_2_PO_4_, 1.3 MgSO_4_, 26 NaCO_3_, 2.4 CaCl_2_ and 10 glucose at room temperature until used for recording. Electrophysiological recordings: After at least 1 h of equilibrium, one slice was transferred to an interface recording chamber and perfused with aCSF at a rate of 1.5–2 ml/min, with the surface of slices exposed to warm, humidified 95% O_2_-5% CO_2_. Field EPSPs (fEPSPs) were recorded from stratum radiatum of CA1 using a glass pipette filled with 2M NaCl (yielding a resistance of 2–3 MΩ) in response to orthodromic stimulation (twisted nichrome wires, 50 µm) of the Schaffer collateral-commissural projections in CA1 stratum radiatum. Pulses of 0.1 ms duration were delivered to the stimulating electrode every 20 sec. The responses were amplified with Axoclamp 2A DC amplifier (Axon Instruments, Foster City, CA), filtered at 6 kHz and digitized at 20 kHz. Data acquisition was controlled by Clampex 9.0 software (Axon Instruments, Foster City, CA). Input/output (I/O) curves were generated using stimulus intensities from 100–350 µA in increments of 50 µA. Baseline fEPSP were evoked at 30-50% of maximal fEPSP in 20 sec intervals. LTP was induced at baseline intensity using Theta Burst Stimulation (TBS) consisting of ten trains of five 100Hz stimulation repeated at 5 Hz. Recording continued for at least 30 min following TBS. fEPSP slope magnitude was calculated as the difference between two cursors, separated by 1 ms, and placed on the middle portion of the ascending phase of the fEPSP. Three consecutive responses separated with 20 sec intervals were averaged and presented as a single point to reduce deviations. LTP were expressed as a percentage of the average slope from the baseline recordings. Comparison of theta burst-induced plasticity was performed between groups using repeated measures ANOVA (across all post-theta burst time points). This analysis was followed by a post-hoc t-test performed between groups for the average change in fEPSP amplitude recorded during the final 5 minutes of recording (35-40 min post theta burst stimulation).

### Data analysis

Student’s two-tailed t-test was used for statistical analysis of paired data. The level of statistical significance and the values of *n* are indicated in the respective figures. ANOVA- Groups were initially compared for differences in tetanus-induced plasticity by performing a repeated measures analysis of variance (ANOVA) across the entire post-tetanus sampling period.

## Results

### Effect of lipoic acid administration on brain glucose uptake

**Figure 1:**
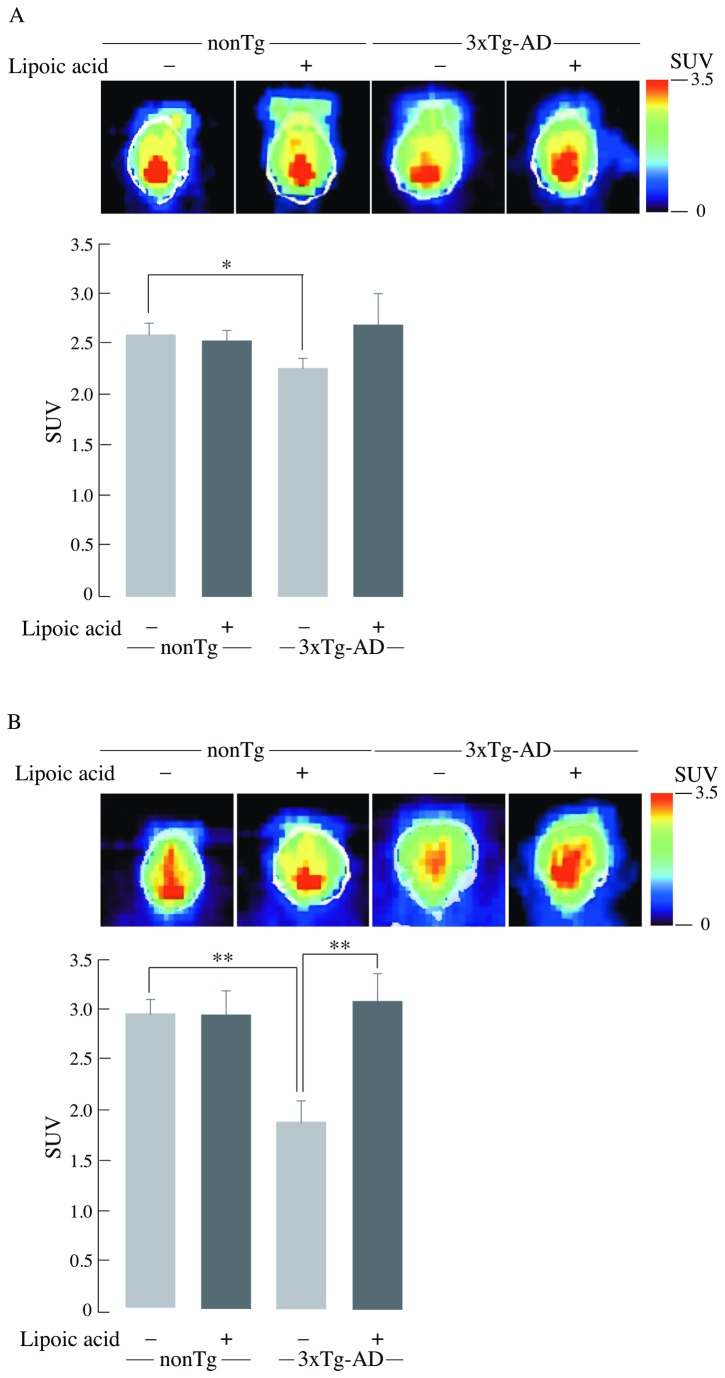
Age-dependent decrease of whole brain glucose uptake and the restorative effect of lipoic acid. Standard uptake value (SUV) was calculated after [^18^F]-FDG injection followed by PET and CT scanning as described in the Materials and Methods section. (A) Young mice, *n* = 34, *n* ≥ 6/group. (B) Old mice, *n* = 27, *n* ≥ 6/group. Upper panel: Representative combined images from PET-CT scanning of nonTg and 3xTg-AD mice ± lipoic acid; lower panel: Average SUV values with the error bar indicating ± SEM. *P ≤ 0.05, **P ≤ 0.01.

[^18^F]-FDG-PET imaging (dynamic microPET scanning) revealed a slight decline in glucose uptake of the young (6-month old) 3xTg-AD compared to age matched nonTg mice (Figure 1A) (SUV_3xTg-AD_ = 2.2 versus SUV_non-Tg_ = 2.6; p ≤ 0.05). A prominent difference in glucose uptake was found in the older (12 month-old mice) 3xTg-AD compared to nonTg mice (Figure 1B) (SUV_3xTg-AD_ = 1.8 versus SUV_non-Tg_ = 2.9; p ≤ 0.01). This suggests that deregulation of glucose uptake occurs at a younger age in the 3xTg-AD mice and that it increases with age. Lipoic acid feeding led to increased brain glucose uptake in young and old 3xTg-AD mice as compared to age-matched 3xTg-AD mice not fed on lipoic acid (Figure 1A and 1B) (SUV_3xTg-AD + lipoic acid_ = 2.7 versus SUV_3xTg-AD_ = 2.2SUV = 2.2; not significant) (SUV_3xTg-AD + lipoic acid_ = 3.1 versus SUV_3xTg-AD_ = 1.8; p ≤ 0.01). This increase in SUV in lipoic acid-fed mice correlated to a net ~20 and ~60% increase of net glucose uptake in lipoic acid fed young and old 3xTg-AD mice, respectively (Figure 1), thus suggesting that lipoic acid can rescue the decreased whole brain glucose uptake.

### Effects of lipoic acid on the total and plasma membrane-associated GLUT3 and GLUT4

**Figure 2:**
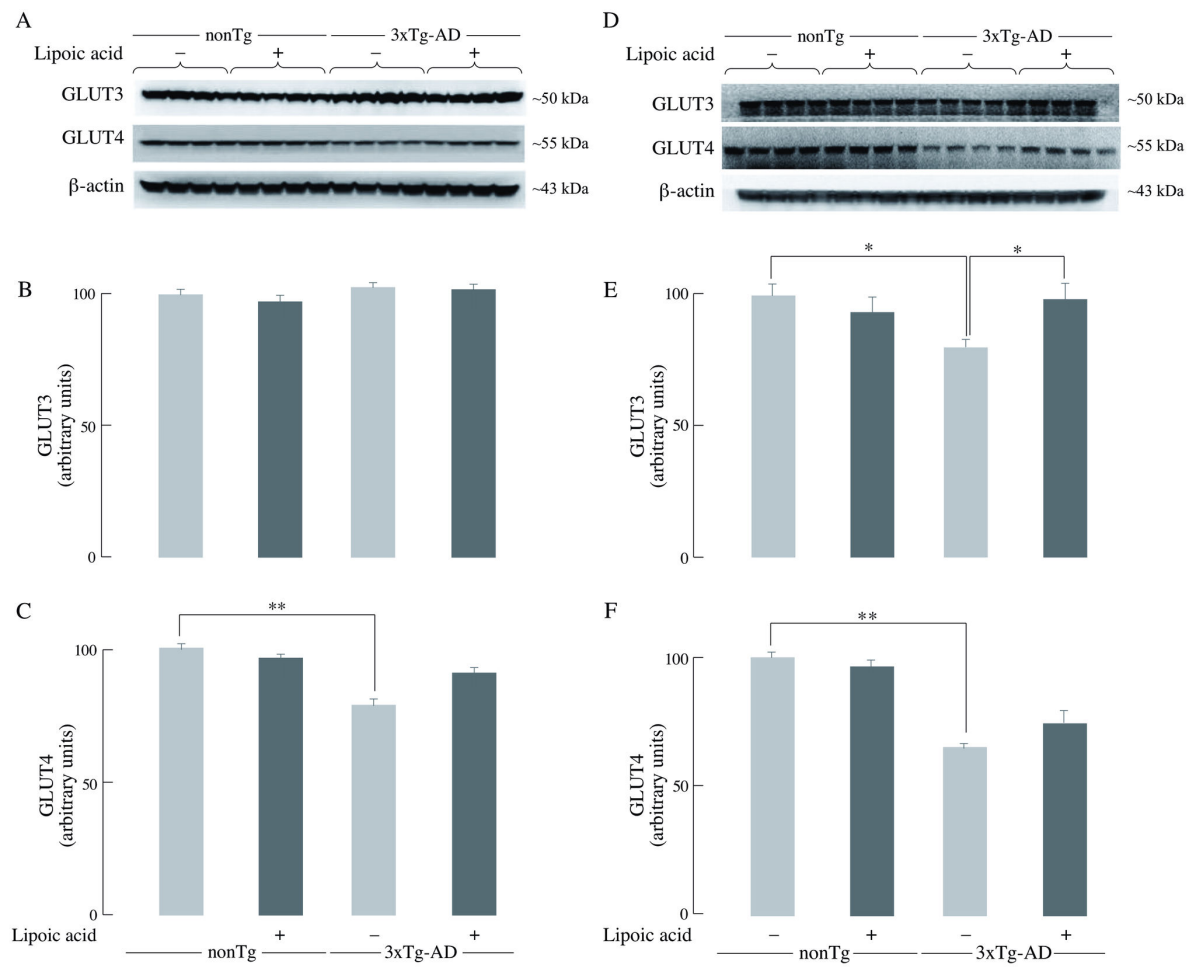
Brain GLUT3 and GLUT4 levels. The levels of total GLUT3 and GLUT4 in whole brain from nonTg and 3xTg-AD mice +/- lipoic acid (young and old) were determined by western-blot analyses. Left panels (A, B, and C) correspond to data from young mice; right panels (D, E, and F) to data from old mice. Representative western blot images of GLUT3, GLUT4, and β-actin (loading control) are shown. Bar graphs show the average GLUT3 or GLUT4 values after normalization with the loading control and the error bars indicating ± SEM. Total *n* = 48, *n* ≥ 5/group. *P ≤ 0.05, **P ≤ 0.01.

The levels of GLUT3 and GLUT4 are critical for glucose transport and its subsequent metabolism to generate energy in neurons. Total GLUT3 levels were not affected in the young 3xTg-AD mice (Figure 2B), whereas old 3xTg-AD mice had significantly lower (~20% decrease) total GLUT3 levels compared to the age matched nonTg mice (Figure 2E). Total GLUT4 was significantly lower by ~20% and ~35% in the young and old 3xTg-AD mice, respectively, as compared to the age-matched nonTg mice (Figure 2C and 2F). Lipoic acid feeding had no significant effect on the total GLUT3 levels of the young nonTg and 3xTg-AD mice (Figure 2B) but it led to a slight increase (~10%) of the total GLUT4 in younger 3xTg-AD mice (Figure 2C). Lipoic acid feeding led to an increase in the total GLUT3 (~20%) (Figure 2E) and GLUT4 (10%) (Figure 2F) in the old 3xTg-AD mice.

**Figure 3:**
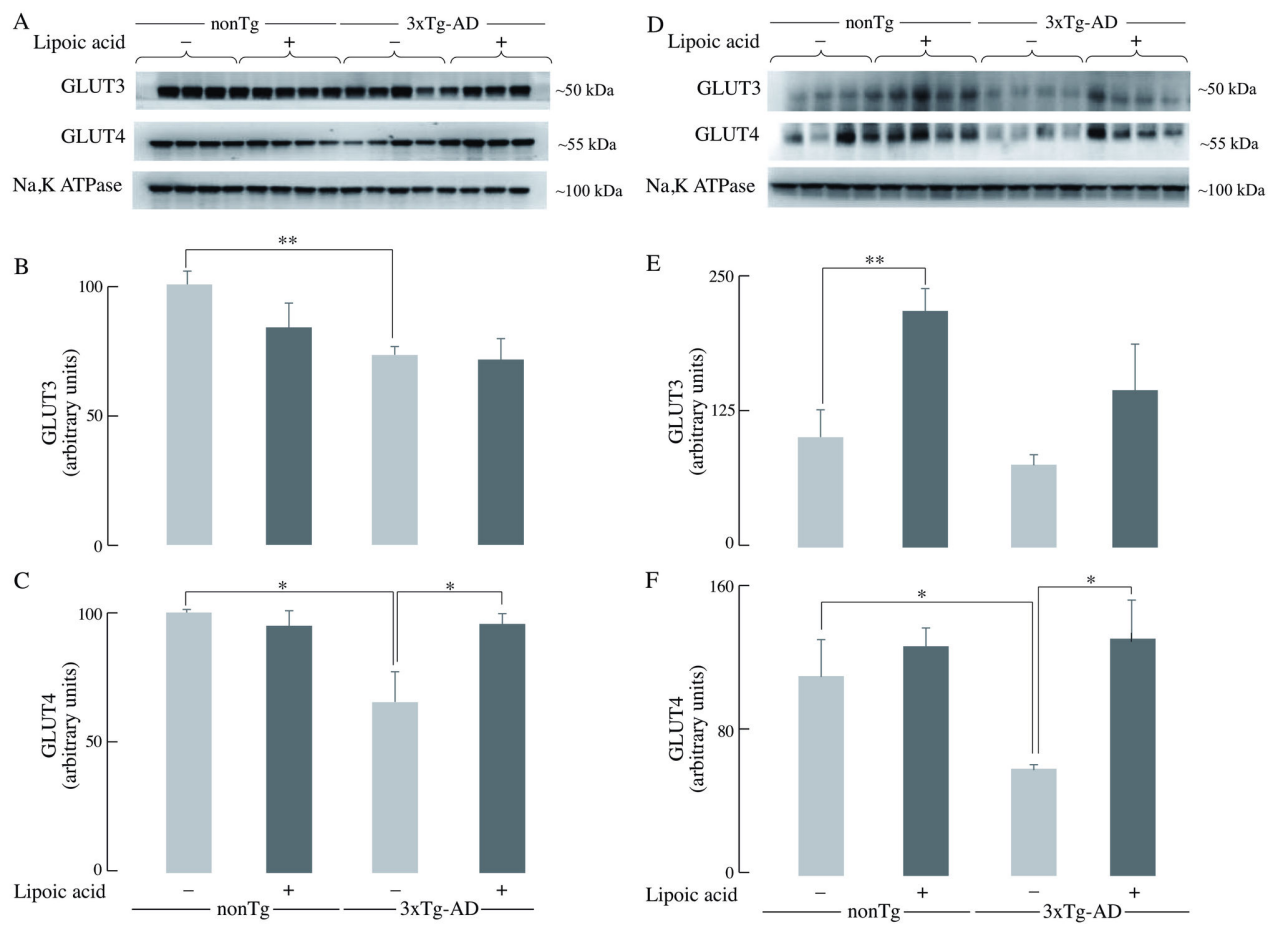
Membrane-associated GLUT3 and GLUT4 levels in brain. The levels of GLUT3 and GLUT4 in whole brain crude membranes from nonTg and 3xTg-AD mice +/- lipoic acid (young and old) were determined by western-blot analyses. Left panels (A, B, and C) correspond to data from young mice, whereas right panels (D, E, and F) correspond to data from old mice. Representative western blot images of GLUT3, GLUT4, and Na, K-ATPase (loading control) in whole brain crude membrane are shown. Bar graphs show the average membrane-associated GLUT3 and GLUT4 values after normalization with the loading control and the error bars indicating ± SEM. Total *n* = 32, *n* = 4/group. *P ≤ 0.05, **P ≤ 0.01.

The total amount of GLUT3 and GLUT4 does not directly indicate the active glucose transporters, for they need to be translocated to the cell surface to facilitate glucose transport in the cell. The amount of plasma membrane-associated GLUT3 was decreased in the young and old 3xTg-AD mice by ~30% as compared to the age-matched nonTg mice (Figure 3B and 3E). The decrease of GLUT4 translocation was particularly drastic, both in the young 3xTg-AD mice (~40% decrease) (Figure 3C) and the old 3xTg-AD mice (~50% decrease) (Figure 3F) as compared to the age- matched nonTg mice. Lipoic acid feeding had no significant effect on the membrane-associated levels of GLUT3 in the young nonTg and 3xTg-AD mice (Figure 3B); however, it led to a ~100% increase in the older nonTg and 3xTg-AD mice (Figure 3E). GLUT4 was increased by lipoic acid feeding in the younger 3xTg-AD mice (~40%) (Figure 3C) but not in the age matched nonTg mice. Among the older mice, lipoic acid feeding led to an increase of GLUT4 in nonTg (~15%) and 3xTg-AD (~115%) mice (Figure 3F).

### Lipoic acid leads to activation of the insulin receptor substrate (IRS)

**Figure 4:**
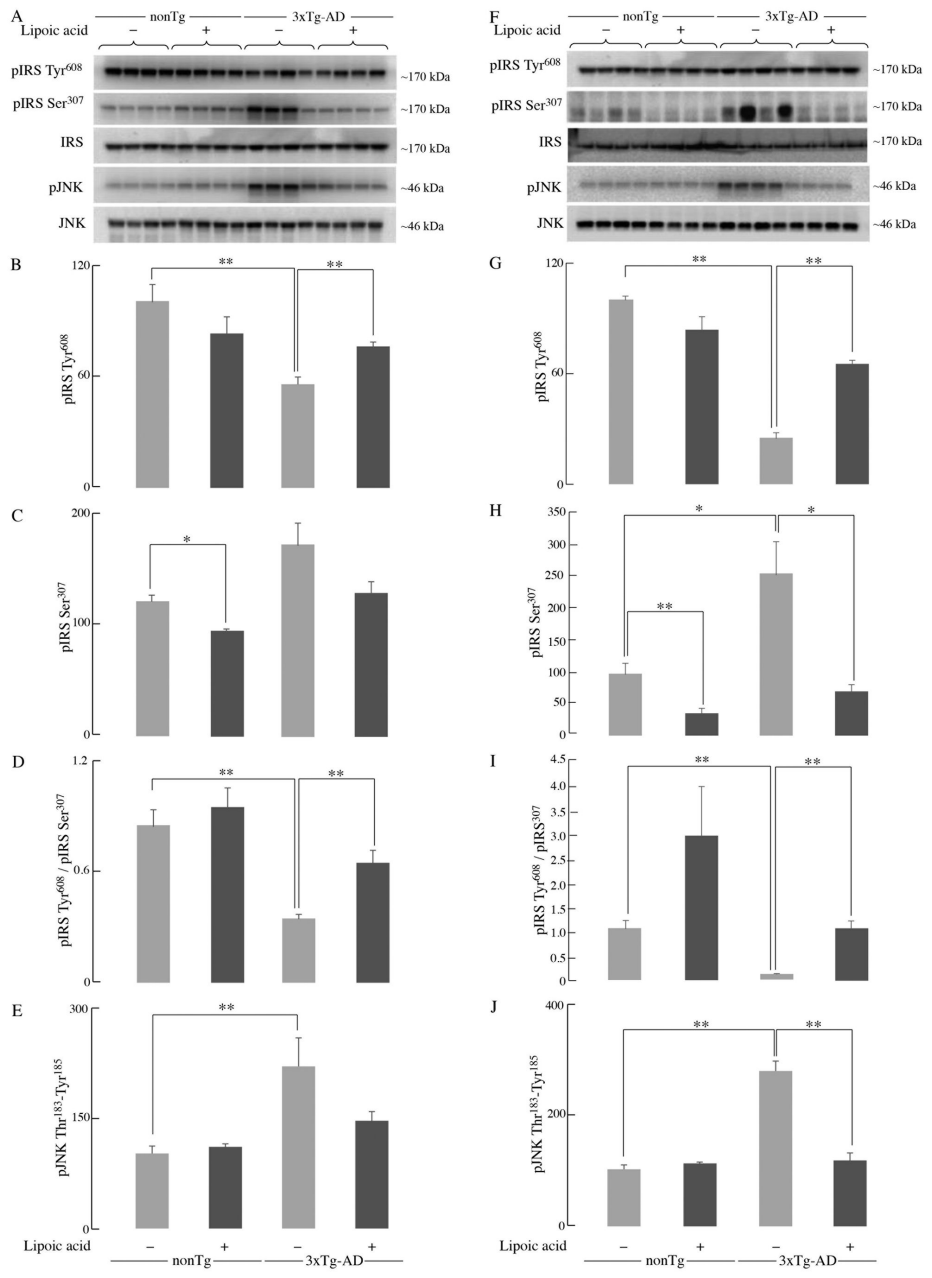
IRS activation status in the 3xTg-AD mice and the effect of lipoic acid. The levels of pIRS-Tyr^608^ (activated) and pIRS-Ser^307^ (inactivated) in whole brain from young and old nonTg and 3xTg-AD mice +/- lipoic acid were determined by western-blot analyses. Left panels (A, B, C, D, and E) correspond to data from young mice; right panels (F, G, H, I, and J) correspond to data from old mice. Bar graphs show the average pIRS Tyr^608^, pIRS Ser^307^, and pJNK Thr^183^-Tyr^185^ values after normalization with the loading control (IRS and JNK) and the error bars indicating ± SEM Total *n* = 48, *n* ≥ 5/group. *P ≤ 0.05, **P ≤ 0.01.

Insulin receptor substrate (IRS) is the immediate downstream substrate of the insulin receptor after activation of the latter. One of the major effects of IRS activation is the downstream activation of the Akt pathway through phosphatidylinositol 3-kinase (PI3K) [30]. Young 3xTg-AD mice showed ~45% decrease in the phosphorylation of IRS on the Tyr^608^ residue compared to age matched nonTg mice (Figure 4B), whereas old 3xTg-AD mice showed a prominent (~75%) decrease of pIRS Tyr^608^ phosphorylation (Figure 4G). Lipoic acid feeding did not lead to a statistically significant difference of pIRS Tyr^608^ phosphorylation among the young nonTg; however, it elicited a ~35% increase of pIRS Tyr^608^ phosphorylation among the young 3xTg-AD mice (Figure 4B). Lipoic acid feeding was found to increase this phosphorylation substantially by ~2.5 fold in the old 3xTg-AD mice (Figure 4G). A slight, not statistically significant decrease of pIRS Tyr^608^ phosphorylation was found in the young and old nonTg mice supplemented with lipoic acid (Figure 4B and 4G).

Tyrosine phosphorylation of IRS-1 leads to its activation, whereas, phosphorylation on the Ser^307^ residue leads to its inactivation. The serine phosphorylation, and thus the IRS-1 inactivation, is associated with c-Jun NH_2_-terminal Kinase (JNK) activation [31,32]. Young and old 3xTg-AD mice showed an increase in Ser^307^ phosphorylation compared to age-matched controls. The younger 3xTg-AD mice showed an increase of ~40% (Figure 4C), whereas the older showed an increase of 150% (Figure 4H). Lipoic acid feeding was clearly effective in reducing the Ser^307^ phosphorylation mediated IRS-1 inactivation as it decreased it by ~20% (Figure 4C) and ~65% (Figure 4H) in the young and old nonTg mice respectively. In the young and old 3xTg-AD mice, lipoic acid feeding led to a decrease in Ser^307^ phosphorylation by ~25% and ~70% respectively (Figure 4C and 4H). Because active JNK (bisphosphorylated) is mainly responsible for the serine phosphorylation of IRS-1, the status of JNK1 phosphorylation in these mice was assessed and found its activation to be in agreement with the IRS-1 serine phosphorylation data. The levels of pJNK1 phosphorylated at Thr^183^–Tyr^185^ (associated with its activation) in the 3xTg-AD mice were increased by ~ 2 fold and ~ 2.8 fold in the young and old mice, respectively, as compared to the age-matched nonTg mice. Lipoic acid supplementation decreased the levels of pJNK in both young and old 3xTg-AD mice (Figure 4E and 4J).

### Lipoic acid mediated activation of the Akt signaling

**Figure 5:**
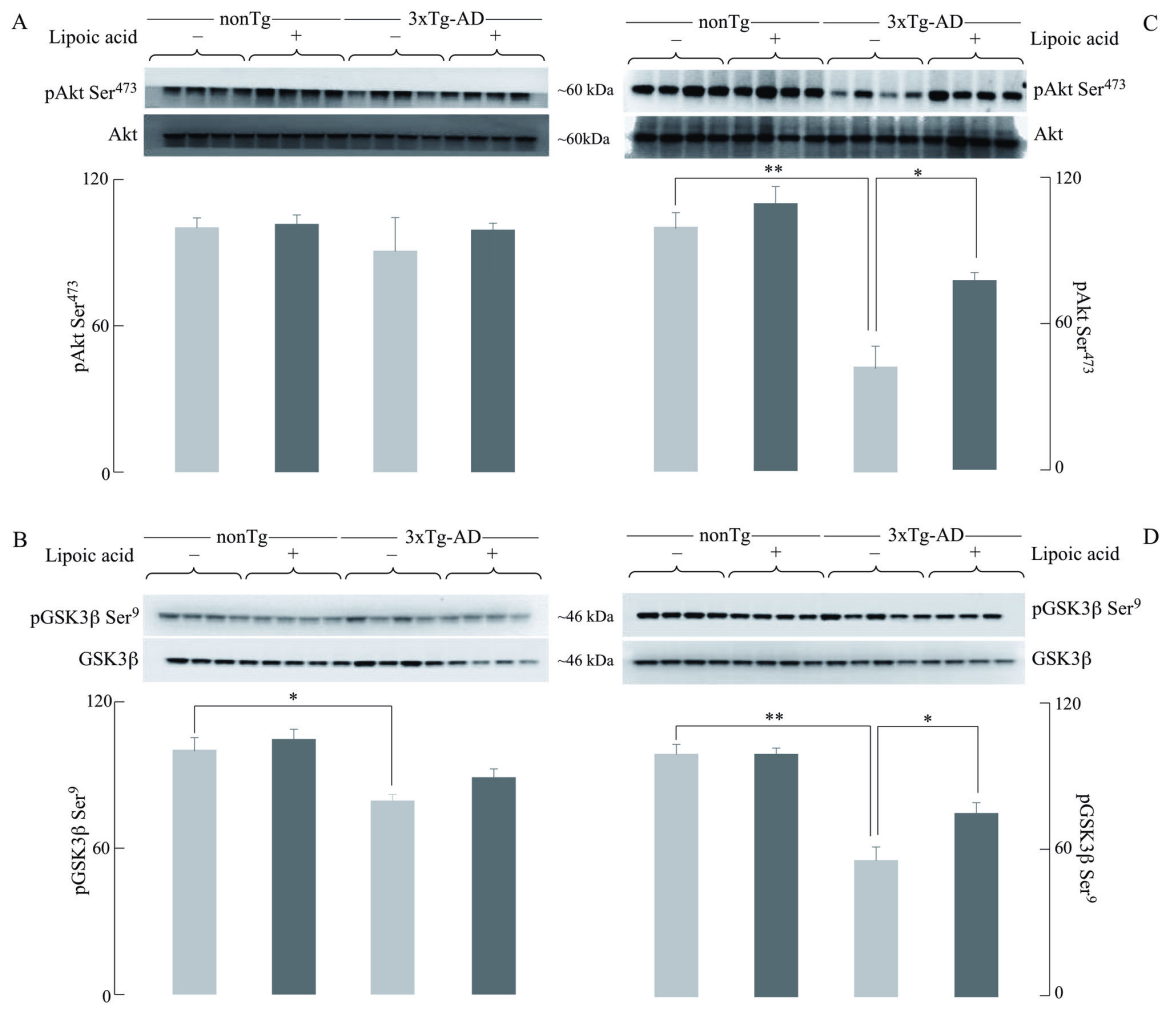
Effect of lipoic acid on age-dependent changes in brain pAkt and pGSK3β. Western blot analyses of the levels of pAkt Ser^473^ and pGSK3β Ser^9^ in whole brain from nonTg and 3xTg-AD mice +/- lipoic acid. Left panels (A and B) correspond to data from young mice and right panels (C and D) to data from old mice. Bar graphs show the average pAkt Ser^473^ (normalized to loading control, Akt) and pGSK3β Ser^9^ (normalized to loading control, GSK3β) with error bars indicating ± SEM. Total *n* = 48, *n* ≥ 5/group. *P ≤ 0.05, **P ≤ 0.01.

 Akt activation, through phosphorylation on Thr^308^ and Ser^473^, leads to the translocation of GLUT3 and GLUT4 to the plasma membrane, thus facilitating glucose transport [33,34]. Western blotting for activated Akt (phosphorylated on Ser^473^) showed that there was no substantial difference among the young nonTg and 3xTg-AD mice, and among those treated with lipoic acid (Figure 5A). However, there was ~60% decrease of Akt phosphorylated at Ser^473^ in the older 3xTg-AD mice compared with the age-matched nonTg (Figure 5C). Moreover, feeding lipoic acid increased activated Akt by ~10% and ~40% in the old nonTg and 3xTg-AD mice, respectively (Figure 5C).

An important downstream target of activated Akt is GSK-3β, phosphorylated (and inactivated) at Ser^9^ (46). GSK-3β is widely implicated in several cellular pathways and, in the context of Alzheimer’s disease, associated with the hyperphosphorylation of the microtubule-associated protein tau [35]. The extent of GSK-3β phosphorylated at Ser^9^ decreases in the 3xTg-AD mice compared to the nonTg mice. Young 3xTg-AD mice show ~20% less phosphorylation (Figure 5B), whereas older 3xTg-AD mice show ~50% lesser phosphorylation (Figure 5D) when compared to the nonTg mice of the same age. Lipoic acid feeding to the young and old 3xTg-AD mice lead to increased phosphorylation of GSK3β (thus, increasing the extent of inactivation): a slight increase in the young 3xTg-AD mice (Figure 5B) and a substantial increase in the older mice (~35%) (Figure 5D). These results are consistent with those observed in terms of Akt phosphorylation at Ser^473^.

### Effect of PI3K/Akt signaling on neuronal energy metabolism

**Figure 6:**
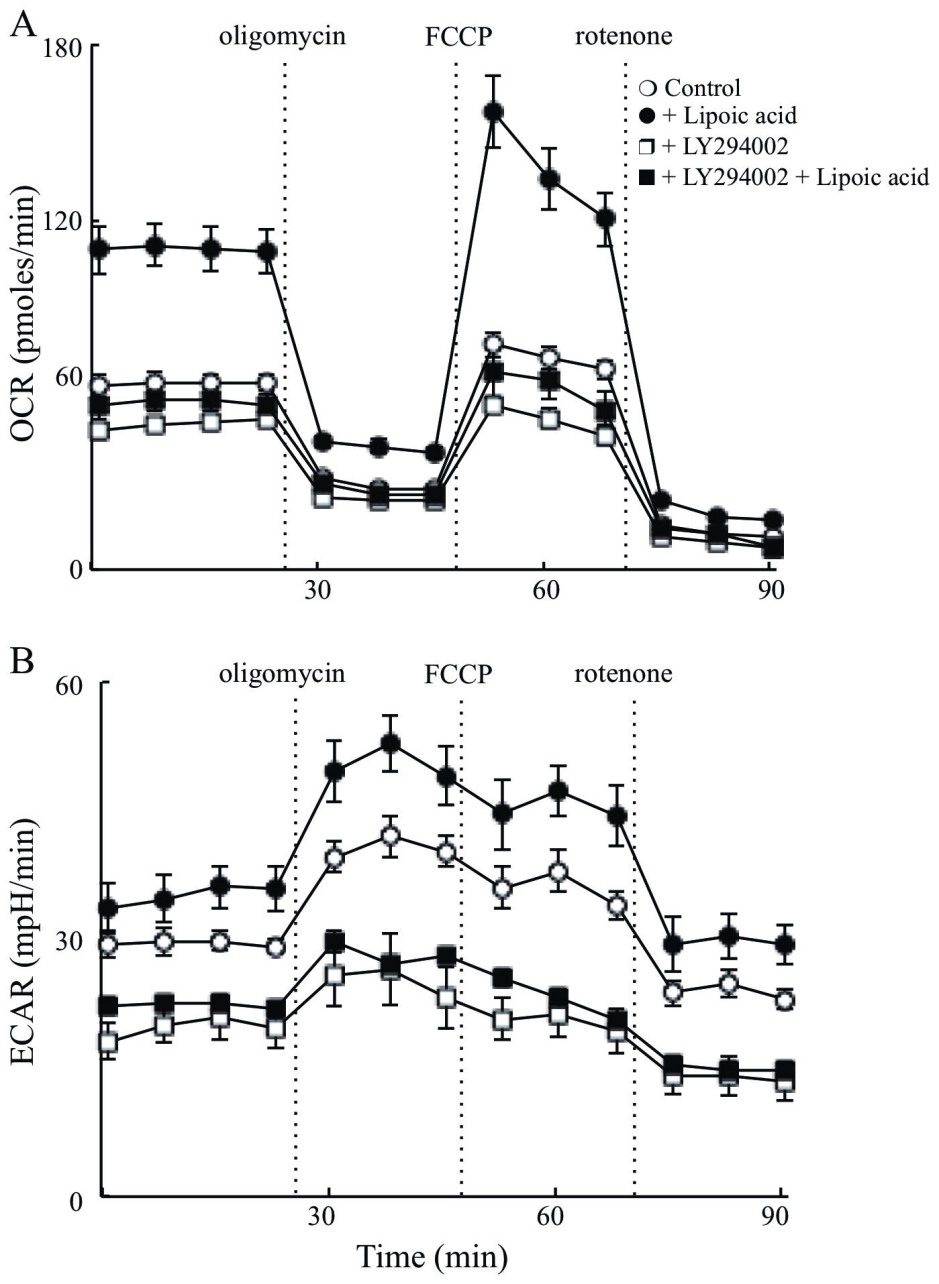
PI3K dependent effect of lipoic acid on cellular bioenergetics. Primary cortical neurons from nonTg mice were isolated and cultured for 7 days. 18 hours before the assay, lipoic acid (20 µM) and/or LY294002 (50 µM) were added to medium. (A) OCR and (B) ECAR were determined using Seahorse XF-24 Metabolic Flux Analyzer. Vertical dashed lines indicate time of addition of mitochondrial inhibitors: oligomycin (4 µM), FCCP (1 µM), and rotenone (1 µM) (open circles). control; (closed circles) plus lipoic acid; (open squares) plus LY294002; (closed squares) plus lipoic acid and LY294002. OCR and ECAR readings were normalized to total protein concentration in each well.

The oxygen consumption rate (OCR) and extracellular acidification rate (ECAR) of primary cortical neurons of nonTg mice was assessed with the extracellular flux analyzer. This approach permits gaining mechanistic insights as to the site of lipoic acid action. OCR represents measurements of mitochondrial respiration (i.e., metabolism of glucose to pyruvate and pyruvate metabolism in mitochondria), whilst ECAR measures extracellular acidification and is indicative of glycolysis (i.e., lactate formation). Lipoic acid pre-treated neurons showed a substantially increased basal OCR by ~2 fold and stimulated ATP turnover, maximal respiratory capacity, and reserve respiratory capacity (Figure 6A and Table 1) as compared to un-treated neurons. Inhibition of PI3K by LY294002 resulted in a slight decrease in basal OCR but the inhibitor abolished the lipoic acid-mediated effect (Figure 6A), thus suggesting that lipoic acid action was upstream of PI3K. The increase in basal ECAR levels was also observed upon treatment with lipoic acid; these effects were abolished by LY294002 (Figure 6B).

**Table 1:** Oxygen consumption rates (OCR) by hippocampal neurons from nonTg mice.

	Control	+lipoic acid	+LY294002	+lipoic acid, +LY294002
Basal respiration	64 ± 3	110 ± 8	50 ± 2	57 ± 4
ATP turnover	35 ± 3	68 ± 8	26 ± 2	30 ± 4
H^+^ leak-induced respiration	29 ± 1	42 ± 2	24 ± 1	27 ± 2
Maximal respiratory capacity	77 ± 4	157 ± 12	57 ± 2	68 ± 8
Non-mitochondrial respiration	13 ± 1	19 ± 1	9 ± 1	11 ± 3
Reserve capacity	13 ± 5	47 ± 15	7 ± 3	10 ± 9

Data expressed in pmoles O_2_/min

### Effects of lipoic acid on Input/Output (I/O)

**Figure 7:**
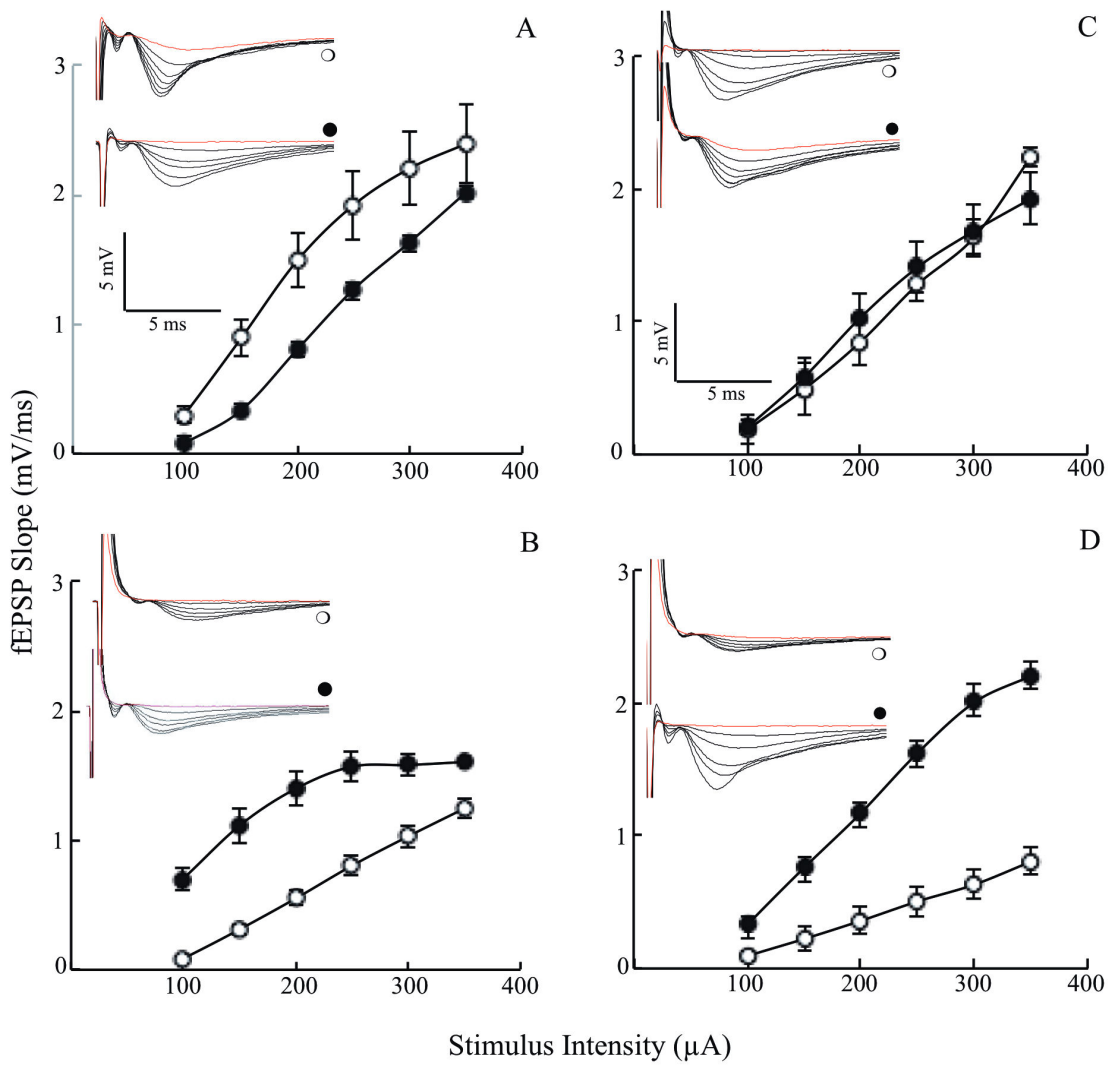
Age dependent changes in I/O of the 3xTg-AD mice and the effect of lipoic acid. I/O relationships after applying increasing stimulation to the stratum radiatum of the CA1 region in the hippocampus for nonTg and 3xTg-AD mice +/- lipoic acid and recording the output (electrophysiology techniques as described in the Materials and Methods section). Left panels (A and B) correspond to data from young mice and right panels (C and D) to data from old mice (open circles). Control; (closed circles) Plus lipoic acid. fEPSP slope (mV/ms) plotted against the corresponding stimulation intensity for (A) young non-Tg mice (*p* < 0.001; F = 27.1 repeated measures ANOVA; young nonTg *n* = 4, young nonTg + lipoic acid *n* = 7); (B) young 3xTg-AD mice (*p* < 0.002; *F* = 20.4 repeated measures ANOVA; young 3xTg-AD *n* = 8, young 3xTg-AD + lipoic acid *n* = 6). (C) old nonTg mice (*p* < 0.004; F = 13.9 repeated measures ANOVA; old nonTg *n* = 6, old nonTg + lipoic acid *n* = 6). (D) old 3xTg-AD mice (*p* < 0.00003; F = 46.4 repeated measures ANOVA; old 3xTg-AD *n* = 6, old 3xTg-AD + lipoic acid *n* = 7). The inserts in each panel are the corresponding representative I/O raw data as obtained during the electrophysiology recordings: (open circles) control and (closed circles) plus lipoic acid. Total *n* = 51 slices, *n* ≥ 5 slices/group and at least 3-4 animals/group.

The data shown above indicated that lipoic acid enhanced glucose uptake (Figure 1) and the translocation to the membrane of GLUT3 and GLUT4 as well as stimulated several components of the PI3K/Akt signaling pathways and increased mitochondrial reserve capacity. However, whether or not lipoic acid treatment enhances function, i.e., synaptic plasticity remains to be determined. Synaptic failure in Alzheimer’s disease has been found to be associated with deficits in numerous neurotransmitters and neurochemicals; these deficits ultimately impair synaptic plasticity and brain function [36,37]. Electrophysiology was employed in this experimental model to measure the I/O responses and gauge the strength of synaptic connections. Young 3xTg-AD mice had smaller synaptic responses compared to age-matched nonTg mice (Figure 7A,B). Lipoic acid feeding was found to have adverse effects in the younger nonTg mice leading to a decrease in the I/O responses (Figure 7A), whereas in the young 3xTg-AD mice, it led to a substantial increase in I/O slopes (Figure 7B). In the 12 month-old mice, the I/O responses were considerably decreased in the 3xTg-AD mice (Figure 7D) as compared to the age-matched nonTg mice (Figure 7C). Lipoic acid feeding had no effect on the older nonTg mice but elicited a substantial increase in I/O responses of the 3xTg-AD mice (Figure 7D).

**Figure 8:**
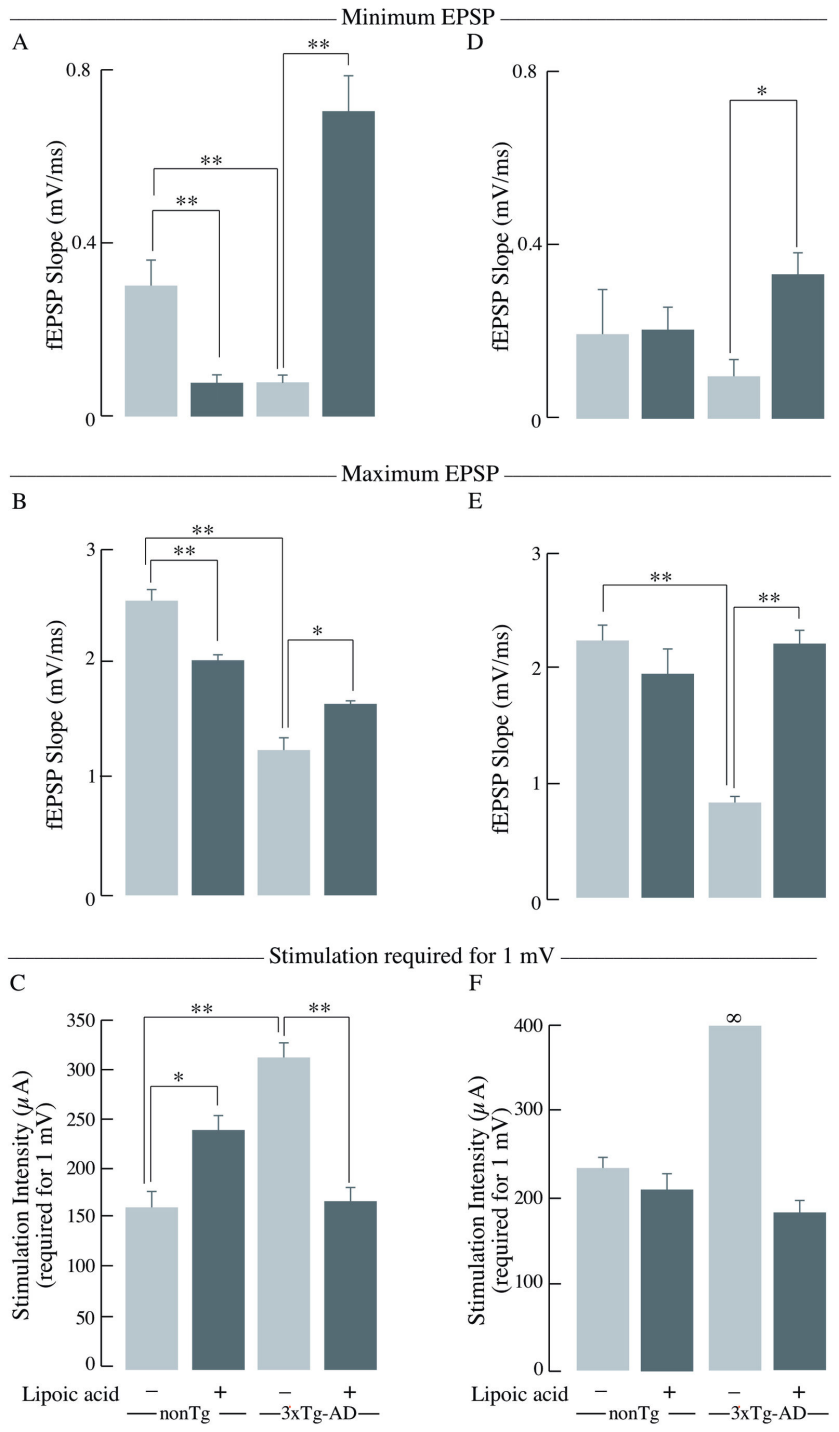
Minimum EPSP, maximum EPSP, and stimulation intensity required to reach 1mV. Bar graphs of the levels of minimum EPSP, maximum EPSP, and stimulation intensity required to reach 1 mV as obtained during the I/O recordings in the stratum radiatum of the hippocampal CA1 region for nonTg and 3xTg-AD mice +/- lipoic acid. Left panels (A, B, and C) correspond to data from young mice and right panels (D, E, and F) to data from old mice. Bar graphs showing the minimum EPSP or the fEPSP slope values at 100 µA and the error bars indicating ± SEM for (A) young mice and (D) old mice. Bar graphs showing the maximum EPSP or the fEPSP slope values at 350 µA and the error bars indicating ± SEM for (B) young mice and (E) old mice. Bar graphs showing the stimulation intensity required to reach at least 1mV output and the error bars indicating ± SEM for (C) young mice (*p* < 0.01; F = 8.9 repeated measures ANOVA) (young nonTg *n* = 7, young 3xTg-AD *n* = 7) and (F) old mice (*p* < 0.003; F = 14.6 repeated measures ANOVA) (old nonTg *n* = 6, old 3xTg-AD *n* = 7). Total *n* = 51 slices, *n* ≥ 5 slices/group and at least 3-4 animals/group. *P ≤ 0.05, **P ≤ 0.01.

In the young 3xTg-AD mice, the minimum and maximum output was significantly decreased compared to the age-matched nonTg mice by ~70% (Figure 8A) and 50% (Figure 8B), respectively. Lipoic acid feeding elicited an increase of the minimum and maximum output in the young 3xTg-AD mice by ~800% and ~30% (Figure 8A,B). Conversely, lipoic acid feeding elicited a decrease in minimum and maximum output in the young nonTg mice (Figure 8A,B). The young 3xTg-AD mice also required considerably greater stimulation intensity to reach a minimum of 1 mV response in comparison with the nonTg mice of the same age (Figure 8C). A similar trend for the minimum and maximum output and stimulation required to reach 1 mV was found for old 3xTg-AD mice (Figure 8D, E, and F). Interestingly, old 3xTg-AD mice failed to reach 1 mV output even at the maximal stimulation intensity, i.e., 350 µA.

### Lipoic acid increases long-term potentiation (LTP) in the old 3xTg-AD mice

**Figure 9:**
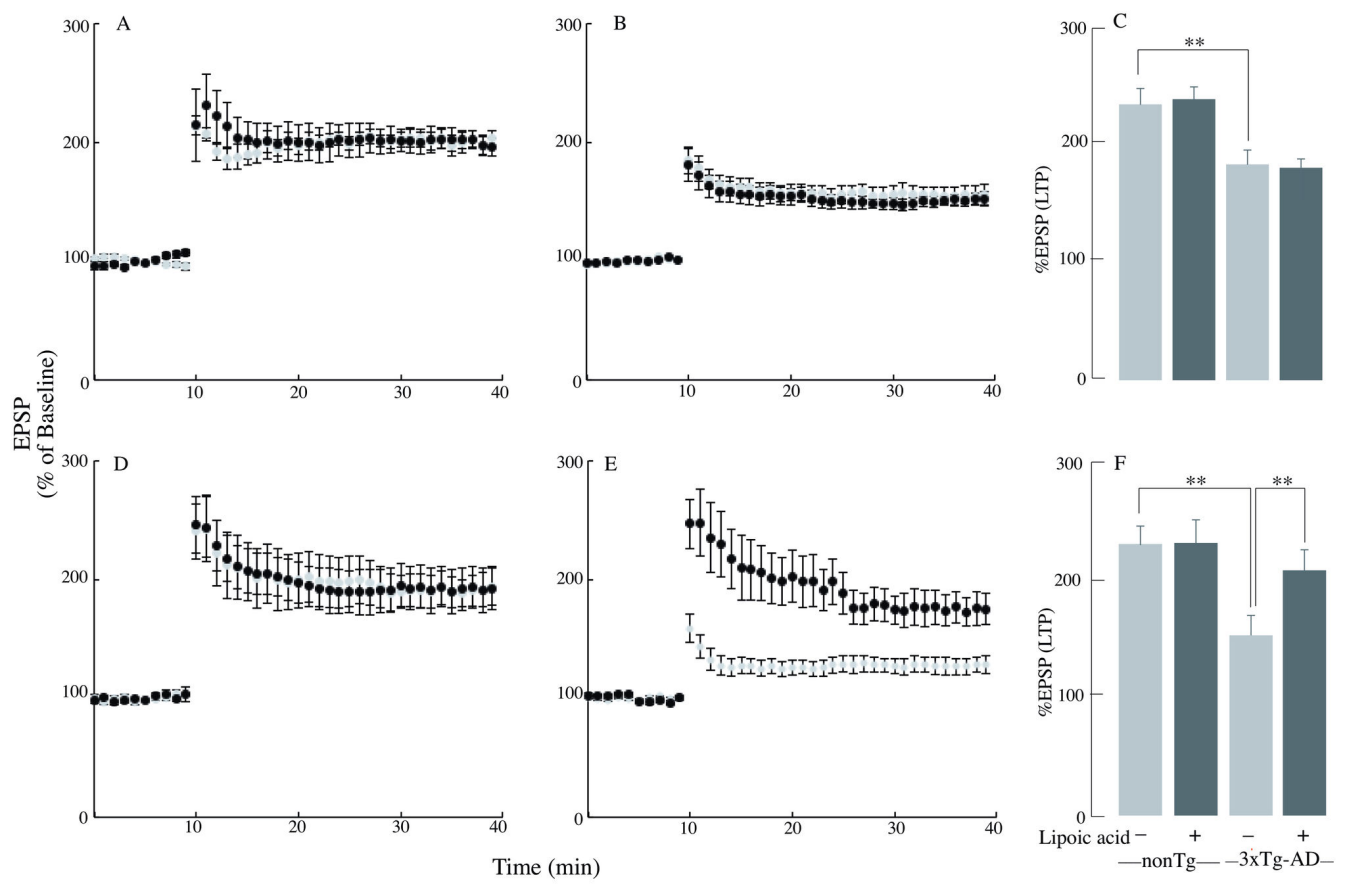
Age dependent changes in the LTP of the 3xTg-AD mice and the lipoic acid effect. LTP was induced at baseline intensity using theta burst stimulation (TBS) consisting of ten trains of five 100 Hz stimulation repeated at 5 Hz. Slope of EPSPs was measured and results normalized to the average value measured during the 10 min baseline period. Recording continued for at least 30 min following TBS and the last 5 min was used to calculate the LTP. Panels A, B, and C correspond to data from young mice, whereas, panels D, E, and F correspond to data from old mice (gray circles/bars – control (nonTg or 3xTg-AD), black circles/bars – fed lipoic acid (nonTg or 3xTg-AD + lipoic acid). A graph showing the first 10 min of baseline followed by the percentage of the baseline response elicited after TBS for 30 min for (A) young nonTg mice, (B) young 3xTg-AD mice, (D) old nonTg mice, (E) old 3xTg-AD mice. Bar graphs showing the measured LTP using % EPSP for the last 5 min of the response to TBS stimulation for (C) young mice and (F) old mice. Total *n* = 51 slices, *n* ≥ 5 slices/group and at least 3-4 animals/group. *P ≤ 0.05, **P ≤ 0.01.

 Long-term potentiation in the CA1 region is widely believed to be a form of plasticity responsible for learning and memory. It involves activation of NMDA receptors for its induction and increased insertion of AMPA (α-amino-3-hydroxy-5-methyl-1,4-isoxazolepropionate) receptors for its expression [22]. The young 3xTg-AD mice expressed reduced LTP (Figure 9B) compared to the nonTg mice (Figure 9A). Lipoic acid feeding had no effect on LTP expression in either young nonTg or 3xTg-AD mice (Figure 9C), which meant that their comparison showed near identical results after lipoic acid feeding.

The old 3xTg-AD mice expressed a substantially reduced LTP (Figure 9E) compared to nonTg mice (Figure 9D). At variance with the results observed in young mice, lipoic acid feeding exerted a profound increase in LTP in the old 3xTg-AD mice (Figure 9E,F). These data show that dietary lipoic acid was not effective in altering the deficits in hippocampal LTP seen in young 3xTg-AD mice, while it was extremely potent in restoring LTP to control levels in 3xTg-AD mice. The old 3xTg-AD mice showed even more reduced LTP than that seen in young 3xTg-AD mice (*p* < 0.04; F = 6.0 repeated measures ANOVA) (old 3xTg AD *n* = 6, old 3xTg-AD + lipoic acid; *n* = 7). However, the improvement in LTP did not bring it to the levels observed in either young nonTg or nonTg-lipoic acid (*p* < 0.05; F = 5.17 repeated measures ANOVA) (young 3xTg AD-lipoic acid *n* = 7, old 3xTg-AD-lipoic acid; *n* = 6).

## Discussion

**Figure 10:**
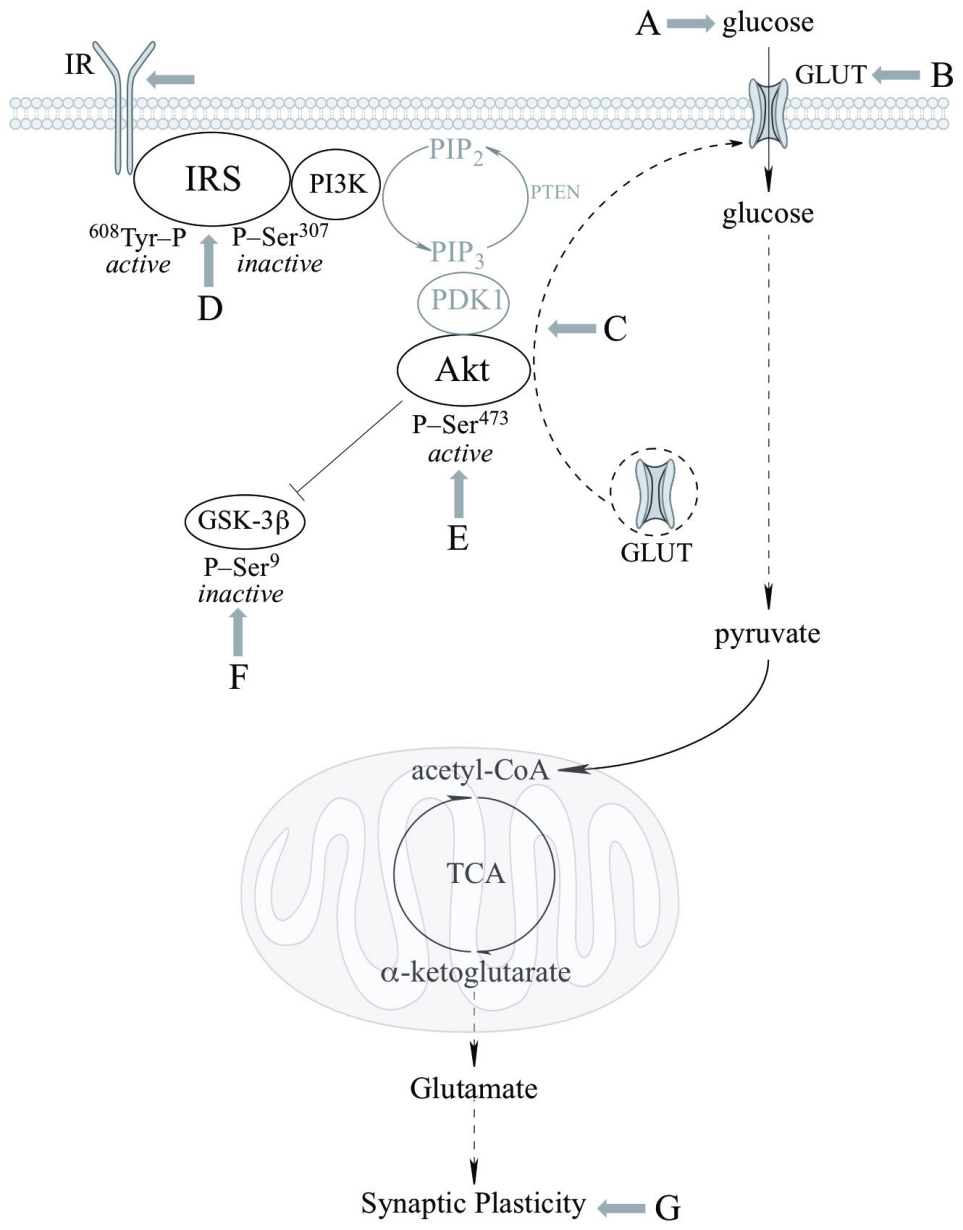
Sites of action and effects of lipoic acid on brain glucose metabolism. The scheme shows the PI3K/Akt pathway of insulin signaling and the effects of lipoic acid on the different components investigated in this study: (A) glucose uptake, (B) total GLUT3 and GLUT4 expression, (C) translocation of GLUT3 and GLUT4 to the plasma membrane from intracellular vesicles, (D) changes in IRS-Tyr^608^ /IRS-Ser^307^ ratio, (E) activation of Akt, (F) phosphorylation of GSK3β at Ser^9^, and (G) synaptic plasticity.

This is a comprehensive study aimed at establishing the effects of dietary lipoic acid on brain function in a triple transgenic mouse model of Alzheimer’s disease and addresses effects on *substrate supply* assessed by brain glucose uptake ([^18^F]-FDG-PET imaging) and glucose transporters translocation to the plasma membrane, the *modulation of glucose metabolism by the PI3K/Akt pathway of insulin signaling*, mitochondrial oxidative metabolism capacity, and *synaptic plasticity*. These effects of lipoic acid are summarized in Figure 10.

Longitudinal studies carried out in human subjects before the onset of clinically diagnosed MCI or Alzheimer’s disease showed a pronounced decrease of brain glucose uptake in individuals who progressed to MCI or Alzheimer’s disease (compared to normal aging individuals) before any clinical diagnosis was possible [13]. This study added an important dimension to earlier studies showing the association of decreased brain glucose uptake in Alzheimer’s disease by suggesting that the accentuated decrease of brain glucose uptake was an early event in the transition from normal aging to MCI and/or to Alzheimer’s disease. Further studies established associations between brain hypometabolism and cognition residing in the parietal and temporal lobar regions in the early stages of the disease and of the frontal regions in the late stages of the disease [38].

Data in the current study showed ~10% decrease in brain glucose uptake in the young 3xTg-AD mice and ~35-40% decrease in the old 3xTg-AD mice, thus suggesting an age-dependent decrease in brain glucose uptake in these mice, thus establishing similarities between the progress of sporadic Alzheimer’s disease and the transgenic 3xTg-AD mouse model used in this study. This further validates the 3xTg-AD mouse model as a tool to study Alzheimer’s disease and the use of PET-CT imaging to follow the progress of MCI and Alzheimer’s disease. Young and old nonTg did not show statistically significant differences in brain glucose uptake. Lipoic acid feeding was able to restore glucose uptake at both ages in the 3xTg-AD mice, however, elicited a more pronounced increase of net glucose uptake in the old mice (Figure 10; component A), possibly due to the significant drop in glucose uptake at that age.

Glucose transport in neurons is facilitated by the different glucose transporters especially, GLUT3 and GLUT4, which are exclusively present in neurons; moreover, GLUT4 is primarily insulin sensitive and is activated after insulin stimulation [39,40,41]. GLUT3 and GLUT4 facilitate glucose transport after they are translocated to the cell surface by appropriate stimulation that involves Akt activation. The decrease of active glucose transporters on the plasma membrane could be the reason for the decrease in glucose uptake as a function of age in the 3xTg-AD mice. Translocation to the plasma membrane of the insulin-sensitive GLUT4 was decreased in young and old 3xTg-AD mice (with greater net drop in the older mice) compared to the age matched nonTg mice; this suggests a decrease in insulin signaling-mediated translocation of GLUT4. GLUT3 showed a slight decrease in the plasma membrane translocation in both young and old 3xTg-AD mice. Total GLUT4 was also decreased in the young and old mice, suggesting a decreased pool of glucose transporters available for translocation to the plasma membrane. Only minor differences were seen in the total GLUT3 at young and old age, thus implying that the capacity for translocation of GLUT3 was not substantially decreased in the 3xTg-AD mice. Lipoic acid feeding lead to increase of both GLUT3 and GLUT4 membrane translocation in the old nonTg and 3xTg-AD mice, demonstrating that lipoic acid facilitates a greater capacity for glucose transport in neurons. Feeding of lipoic acid did not significantly increase the total GLUT3 and GLUT4 in old mice, thus the effect of lipoic acid in increasing total glucose uptake (PET-CT data in Figure 1) might be mainly due to its ability to increase translocation of glucose transporters on the plasma membrane. Overall, brain glucose uptake was decreased in the 3xTg-AD mice and lipoic acid was able to restore the glucose uptake by increasing the translocation of GLUT3 and GLUT4 to the plasma membrane (Figure 10; components B and C). Lipoic acid has previously been shown to increase glucose uptake in L6 muscle cells and 3T3-L1 adipocytes [24,25], induce the redistribution of GLUT4 to the plasma membrane in 3T3-L1 adipocytes [25], and increase insulin sensitivity in diabetic patients [26].

Insulin signaling is responsible for glucose transport in the brain [42] and control of energy homeostasis. Disruption of insulin signaling is expected to affect glucose transport and the subsequent energy generation from glucose oxidation. Samples of autopsied brains from patients with Alzheimer’s disease have shown a deficiency in insulin signaling [43], thus further confirming the link between disrupted insulin signaling and the well-established decrease of brain glucose uptake associated with Alzheimer’s disease. Autophosphorylation of specific tyrosine residues by tyrosine kinase activity of the insulin receptor is considered essential for its activity [44]. IRS phosphorylation at Tyr^608^ was found to ensue after insulin binding to the receptor and is required for the full activation of PI3K [45] that results in phosphorylation of Akt and the ensuing effects on translocation of GLUT4 to the plasma membrane [46] and phosphorylation (inactivation) of GSK-3β. Impaired IRS-1 activation is associated with retarded embroyonic and postnatal growth [47]. In Alzheimer’s disease, wherein loss of neurons is apparent, this cell survival protein is increasingly deregulated or less activated [20].

Data in this study showed that IRS and Akt activation was decreased in the old 3xTg-AD mice with the concomitant activation of JNK; these data might explain the decrease in both brain glucose uptake and GLUT4 translocation to the plasma membrane in old 3xTg-AD mice. An overall reduction of IRS activity in the young 3xTg-AD mice was suggested by the decreased phosphorylation of IRS at Tyr^608^ (activation) and increased phosphorylation of IRS at Ser^307^ (inactivation); however, this could not be explained in lieu of the Akt data i.e., IRS activation was decreased by ~40% in the young 3xTg-AD mice whereas the effect on Akt activation was rather minimal. Conversely, the old 3xTg-AD mice showed a substantial decrease of both - active IRS and Akt. Interestingly, there was ~10 fold difference of active/inactive IRS i.e., pIRS-Tyr^608^/pIRS-S^307^ between the young and old 3xTg-AD mice (Figure 4D and 4I). This provokes a speculation about the occurrence of a threshold for the levels of active/inactive IRS that is required for Akt activation. The increase in brain glucose uptake exerted by lipoic acid feeding poses the question as to whether or not the cyclic disulfide might stimulate insulin signaling: lipoic acid was able to substantially stimulate insulin signaling in the old 3xTg-AD mice, i.e., increase of pIRS-Tyr^608^/pIRS-S^307^, pAkt-Ser^307^, and pGSK3β-Ser^9^ (Figure 10; components D–F). The latter effects are probably a consequence of lipoic acid-mediated thiol/disulfide exchange on IRS followed by an increased phosphorylation at Tyr^608^. This notion is strengthened by the enhancing effect of lipoic acid on OCR in primary cortical neurons and its inhibition by LY294002, suggests that this increase in mitochondrial efficiency is PI3K dependent and the site of action probably upstream of PI3K. This effect of lipoic acid may be accounted for by oxidation of critical cysteine residues in the insulin receptor and IRS [25,48]; this thiol-disulfide exchange mechanism increases the activation of the IRS and it might be surmised that the site(s) of action of lipoic acid might be at the insulin receptor and IRS.

Synaptic strength, measured by I/O, showed significantly lower response in the young 3xTg-AD mice as compared to the young nonTg mice. The older 3xTg-AD mice showed a lower I/O response at all the stimulus intensities and the maximum response is far lower than the older nonTg mice, showing that the strength of synaptic connections is severely affected in the 3xTg-AD mice.

LTP observed in the hippocampal CA1 pyramidal cells has been widely investigated and considered critical for learning and memory. Release of the neurotransmitter glutamate from the presynaptic terminal followed by its binding to the NMDA and AMPA receptors results in depolarization and subsequently expelling of Mg^2+^ [49], thus allowing for Ca^2+^ and Na^+^ to enter the postsynaptic neuron. Ca^2+^ is further believed to trigger several protein kinases and signal transduction cascades involving Ca^2+^/calmodulin-dependent protein kinase II (CaMKII) and protein kinase C (PKC) that ultimately translate the signals to nucleus and initiate the formation of new synapses [22,50]. In addition to kinase activity and signal transduction, protein synthesis follows for maintenance of LTP. Learning and memory are primarily affected in Alzheimer’s disease and thus it was of primary interest to assess LTP in these 3xTg-AD mice and examine the effect that lipoic acid feeding might induce on this machinery of learning and memory. Several Alzheimer’s disease transgenic mouse models have impaired synaptic plasticity, demonstrated by measuring LTP and LTD (using electrophysiology) [29,51,52,53,54]. We examined the effect of the 3xTg-AD genome as well as lipoic acid feeding on the expression of hippocampal LTP induced with TBS. The LTP for the young and older 3xTg-AD mice was found to be lower than the age matched nonTg mice, pointing towards the synaptic deficits. Interestingly, lipoic acid did increase the I/O but had no effect on the LTP of the young 3xTg-AD mice. However, lipoic acid lead to substantial increase in both I/O and LTP of the older 3xTg-AD mice (Figure 10; component G). It may be surmised that dietary lipoic acid that was more effective in reversing the effects that occur later in life in the 3xTg-AD mice, in which a profound deficit in insulin signaling was also observed. Hence, the synaptic plasticity of the older 3xTg-AD mice was substantially decreased and was restored by lipoic acid. Although the mechanistic reasons for the loss/gain of synaptic plasticity in the 3xTg-AD mice remain to be determined, it can be speculated that the activation of insulin signaling via IRS/PI3K/Akt pathway may have increased NMDA receptor conductance and/or AMPA receptor cycling. Previous studies have shown the effect of PI3K/Akt pathway in stimulating LTP [19] and lipoic acid has been shown to reverse the age-related decrease of LTP and memory deficits in aged rats [55,56], to reduce the hippocampal memory deficits in the Tg2576 model of Alzheimer’s disease [57], and to stabilize cognitive functions in patients afflicted with moderate Alzheimer’s disease [58].

## Conclusions

Lipoic acid successfully reverted the age-associated decrease of glucose uptake and stimulated the PI3K/Akt pathway of insulin signaling in the 3xTg-AD mouse model. Importantly, the synaptic deficits associated with the old 3xTg-AD mice were also reversed by lipoic acid feeding. To our knowledge, this is the first study that establishes an age-dependent correlation between the decrease in glucose uptake *in vivo* assessed by PET-CT imaging along with decrease in insulin cell signaling and concomitant decrease of synaptic plasticity in the 3xTg-AD mouse model of Alzheimer’s disease. Albeit, the study does not provide causal relationship between decrease in glucose metabolism and impaired synaptic plasticity, but it hints at a plausible hypothesis that decrease in glucose uptake and metabolism ultimately affects the high energy-demanding synaptic transmission, leading to impaired synaptic plasticity. Previous behavioral studies have shown the positive effects of lipoic acid on memory in different mouse models of aging [59,60,61] and Alzheimer’s disease [57,62]. However, future comprehensive studies on behavioral changes effected by lipoic acid in the 3xTg-AD model are warranted.
