# Correction: Age-Dependent Modulation of Synaptic Plasticity and Insulin Mimetic Effect of Lipoic Acid on a Mouse Model of Alzheimer’s Disease

**DOI:** 10.1371/journal.pone.0116442

**Published:** 2014-12-19

**Authors:** The PLOS ONE Staff

Figure 4 in the published article shows the same Western blot for two different ages of mice in error, in Fig. 4A and Fig. 4F. The representative blot for IRS in Fig. 4A (third from top) and that for pIRS Tyr^608^ in Fig. 4F (first from top) are the same images.

**Figure 4:**
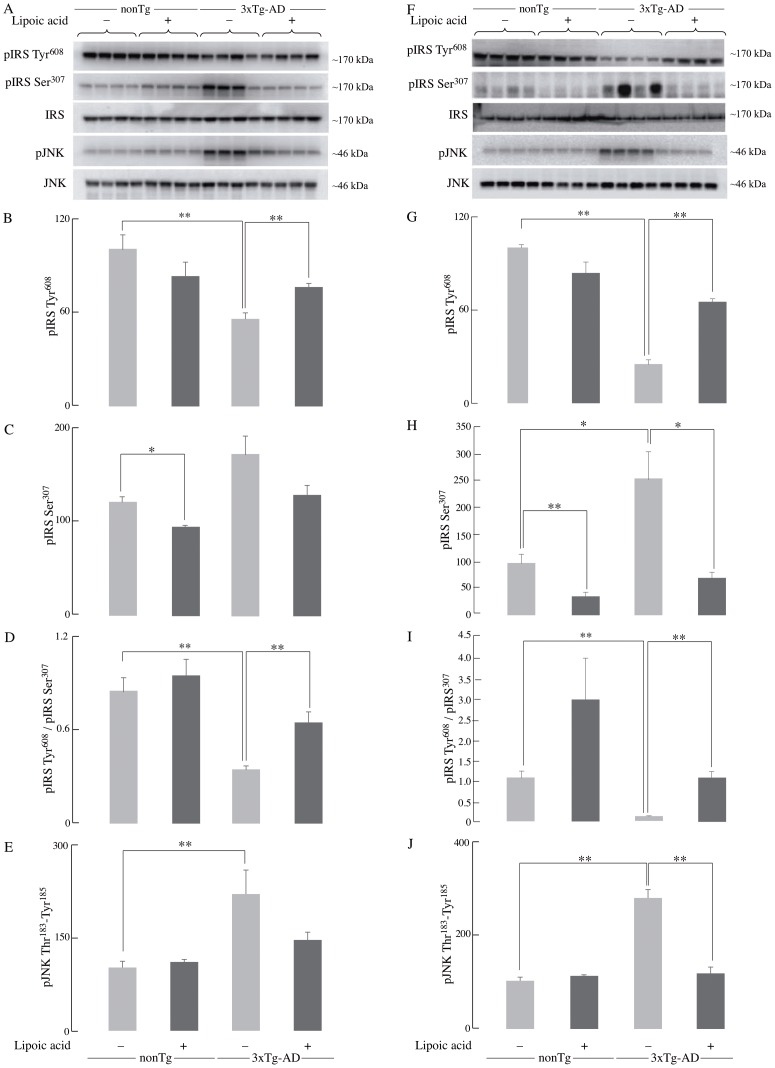
IRS activation status in the 3xTg-AD mice and the effect of lipoic acid. The levels of pIRS-Tyr^608^ (activated) and pIRS-Ser^307^ (inactivated) in whole brain from young and old nonTg and 3xTg-AD mice +/- lipoic acid were determined by western-blot analyses. Left panels (A, B, C, D, and E) correspond to data from young mice; right panels (F, G, H, I, and J) correspond to data from old mice. Bar graphs show the average pIRS Tyr^608^, pIRS Ser^307^, and pJNK Thr^183^-Tyr^185^ values after normalization with the loading control (IRS and JNK) and the error bars indicating ± SEM Total *n*  =  48, *n*≥5/group. *P≤0.05, **P≤0.01.

The pIRS Tyr^608^ in Fig. 4F (first from top) needs to be replaced, and the authors have provided a corrected figure here, as well as the raw blots.

This error was introduced while revising Fig. 4 to add new data during the first revision. The authors apologize for this error. This does not affect the findings in the article, because the mean intensity of the bands was calculated from the correct Western blots.

The authors have provided the original images for Figure 4. These are cropped PVDF membranes. For the older mice another machine was used and the areas of interest were saved.

## Supporting Information

File S1 Original images for Figure 4.
(PPTX)
