# Supplementary material for: Correction: Age-Dependent Modulation of Synaptic Plasticity and Insulin Mimetic Effect of Lipoic Acid on a Mouse Model of Alzheimer’s Disease
Source: PLoS One. 2014 Dec 19;9(12):e116442. doi: 10.1371/journal.pone.0116442 (PMC4272323; doi:10.1371/journal.pone.0116442)
Supplement: File S1 — Original images for Figure 4. (PPTX) [file pone.0116442.s001.pptx]

## Slide 1
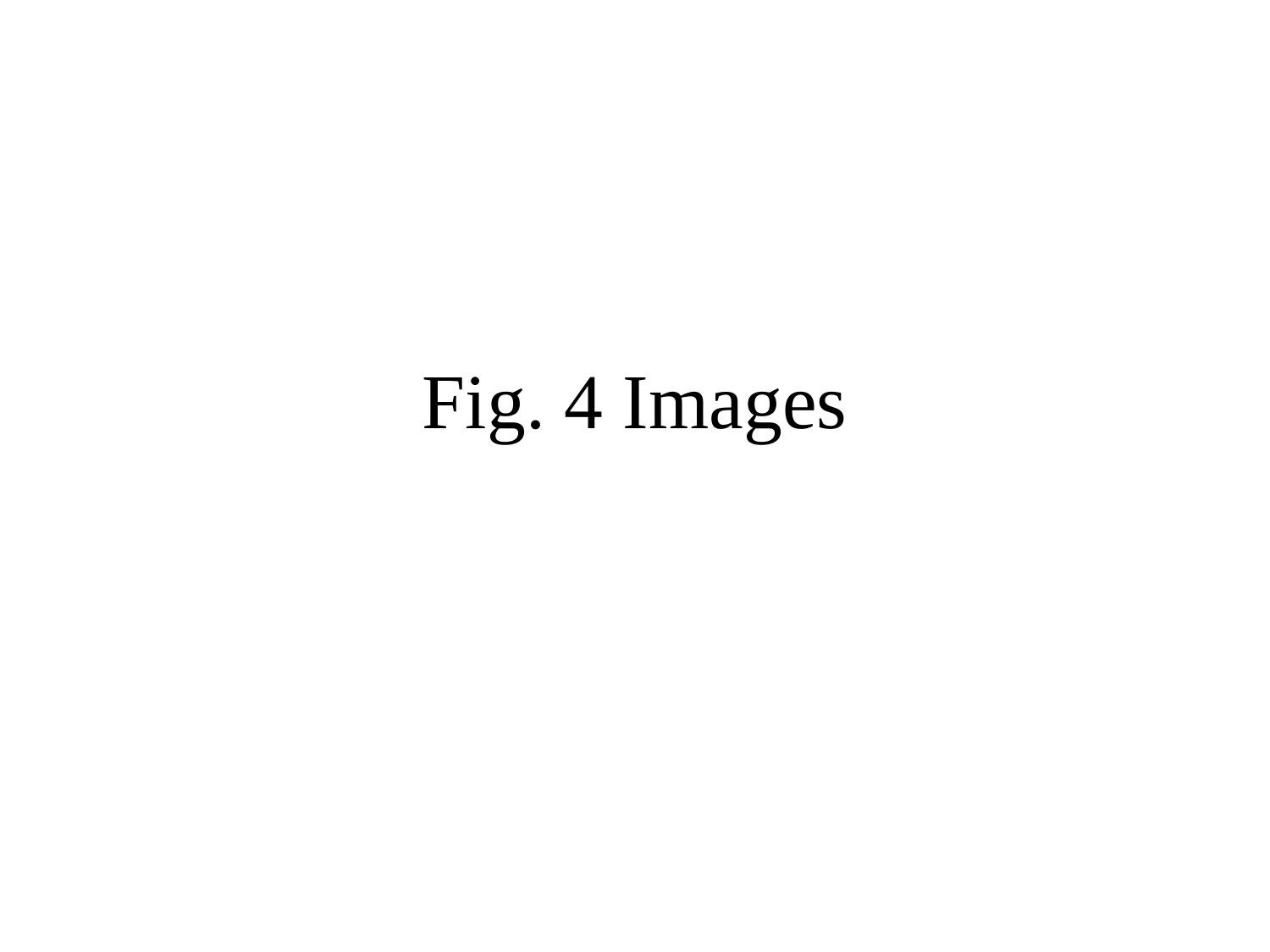

# Fig. 4 Images

## Slide 2
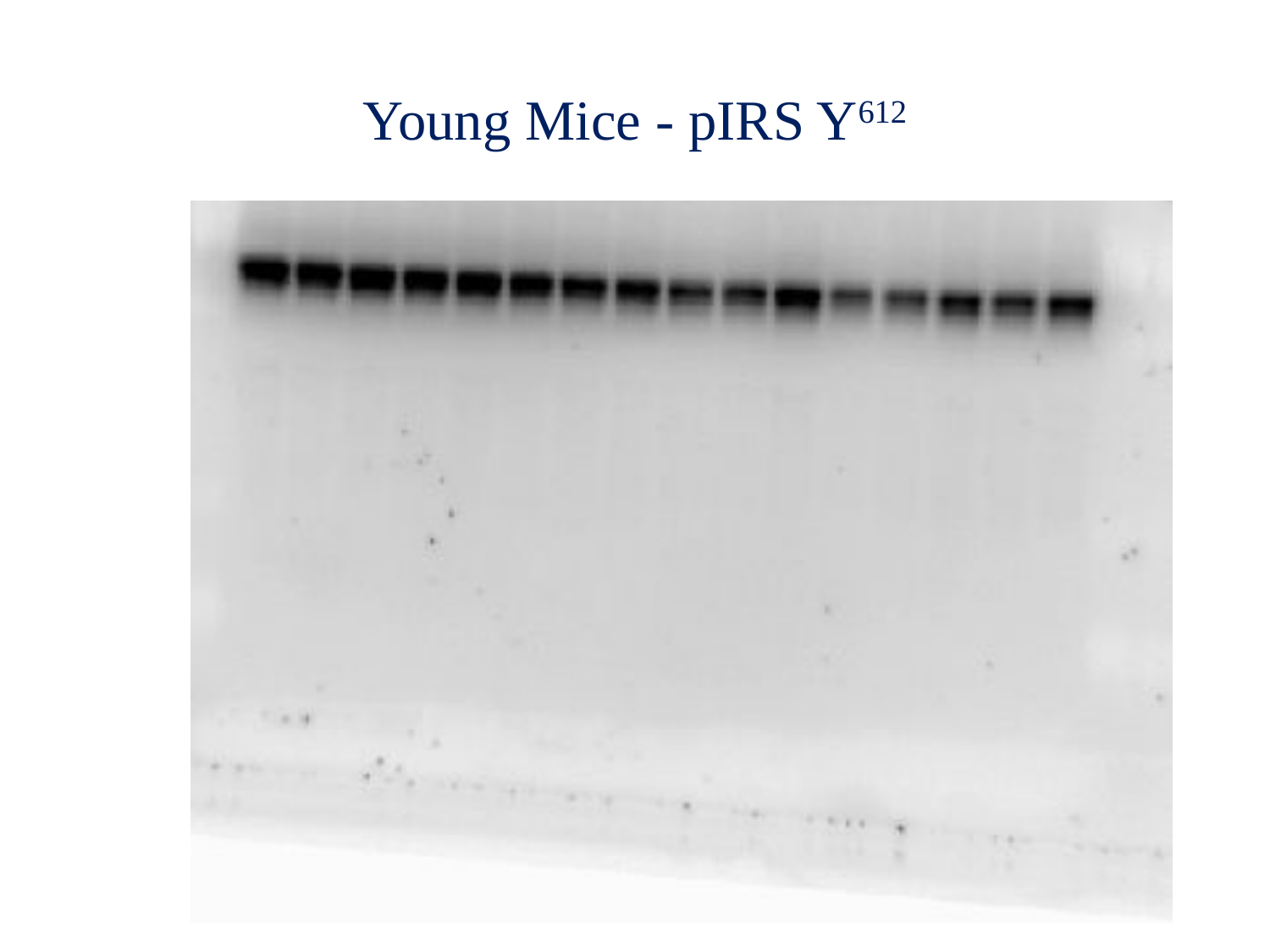

# Young Mice - pIRS Y612

## Slide 3
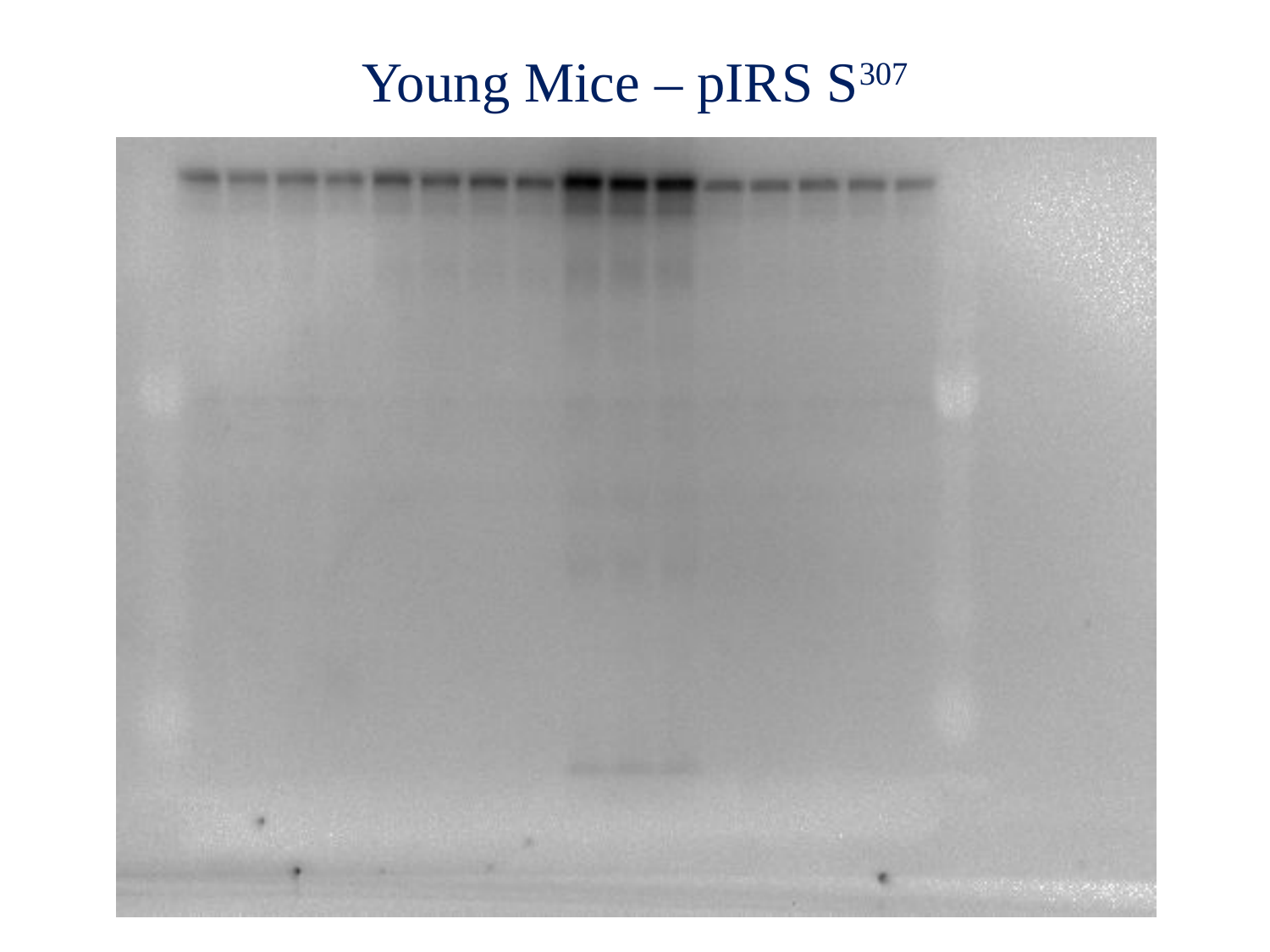

# Young Mice – pIRS S307

## Slide 4
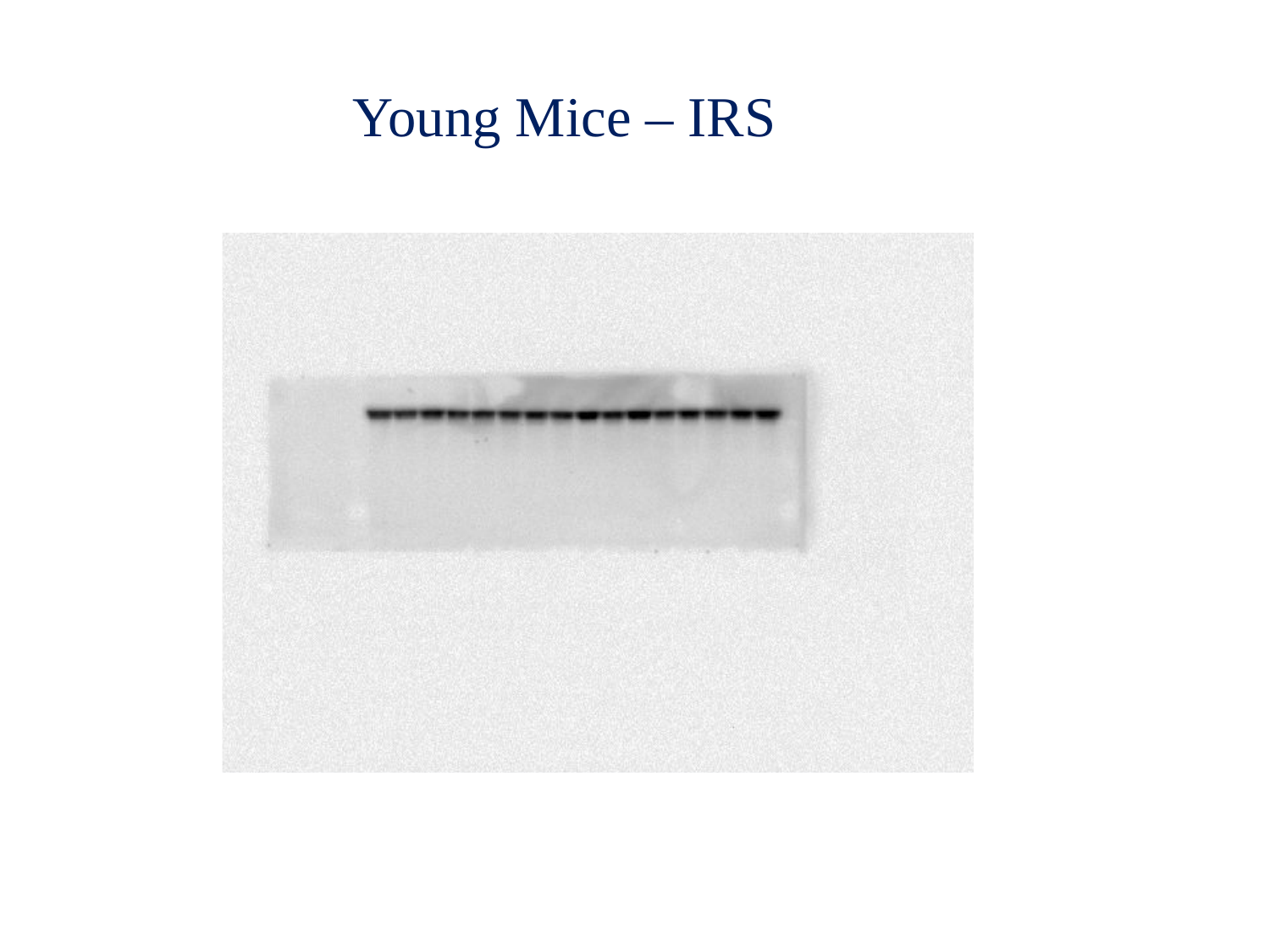

Young Mice – IRS

## Slide 5
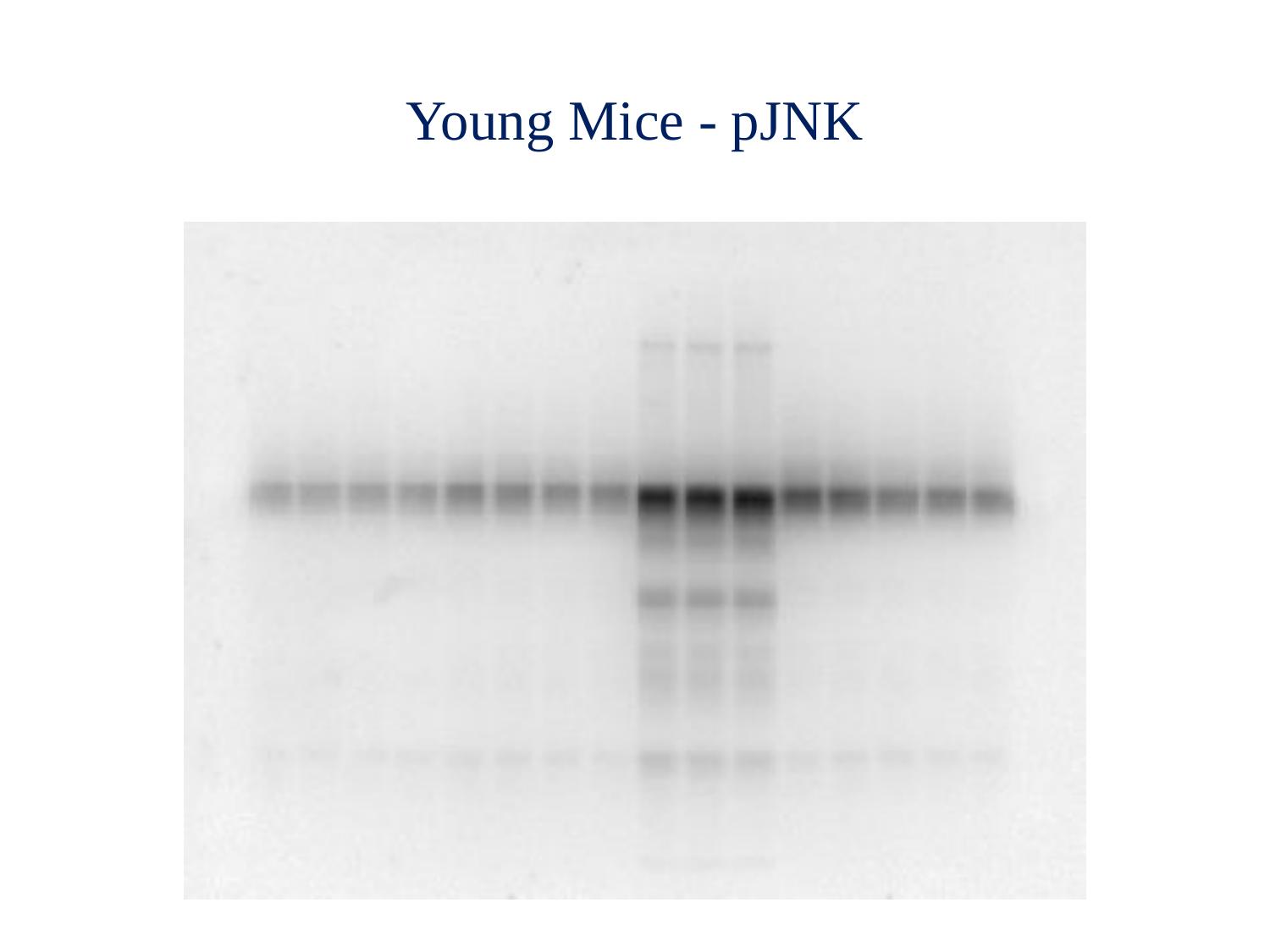

# Young Mice - pJNK

## Slide 6
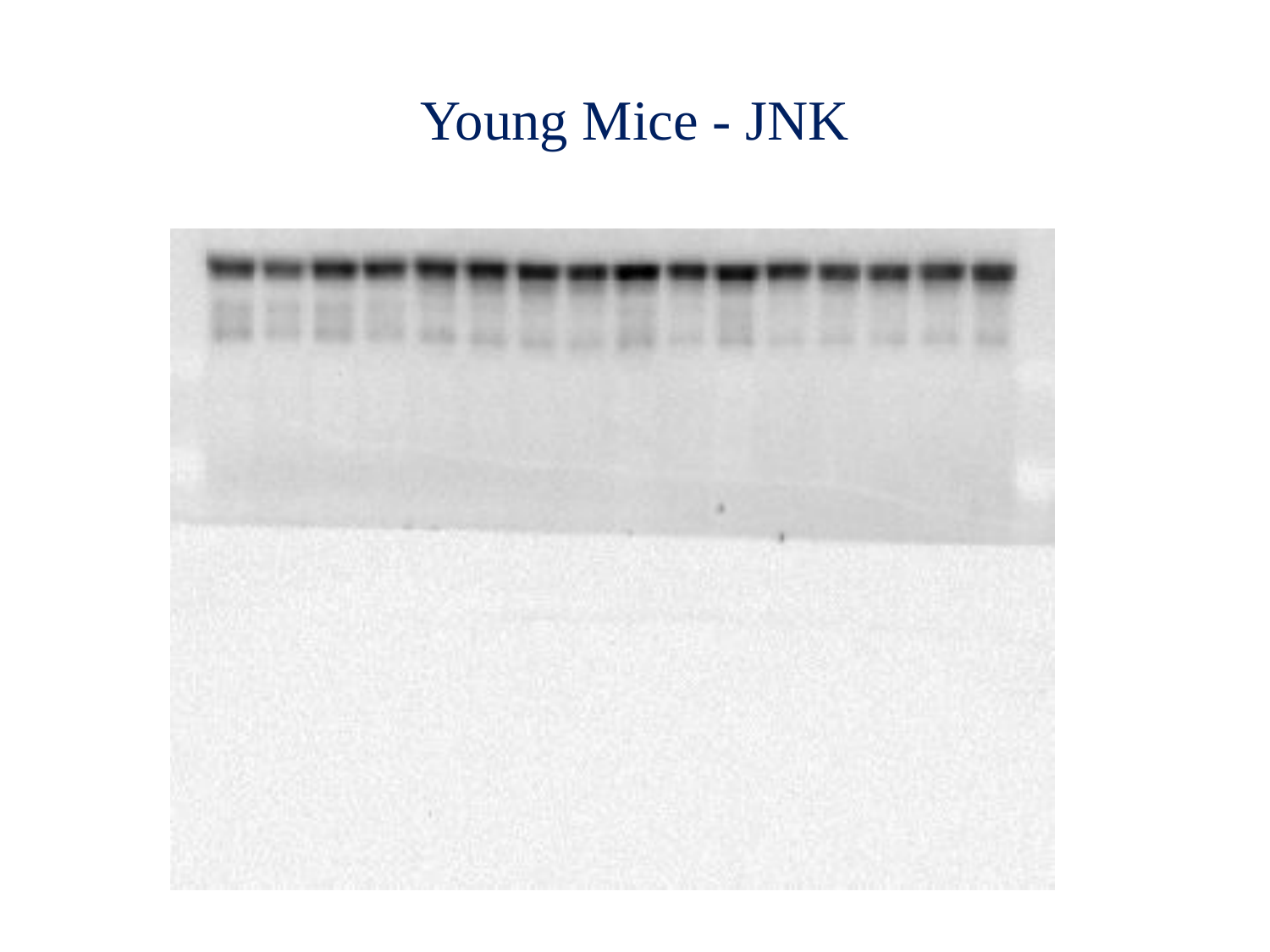

# Young Mice - JNK

## Slide 7
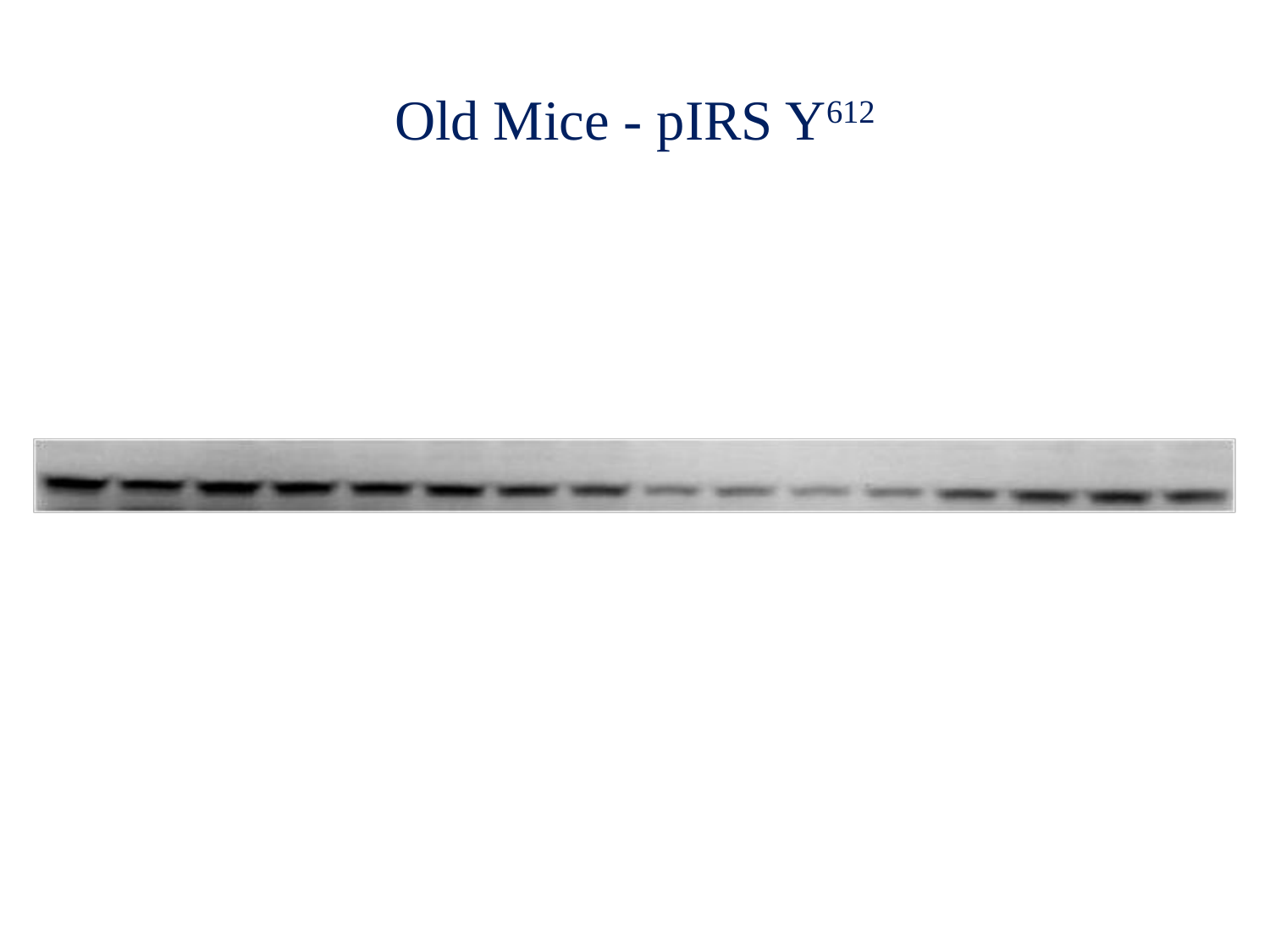

# Old Mice - pIRS Y612

## Slide 8
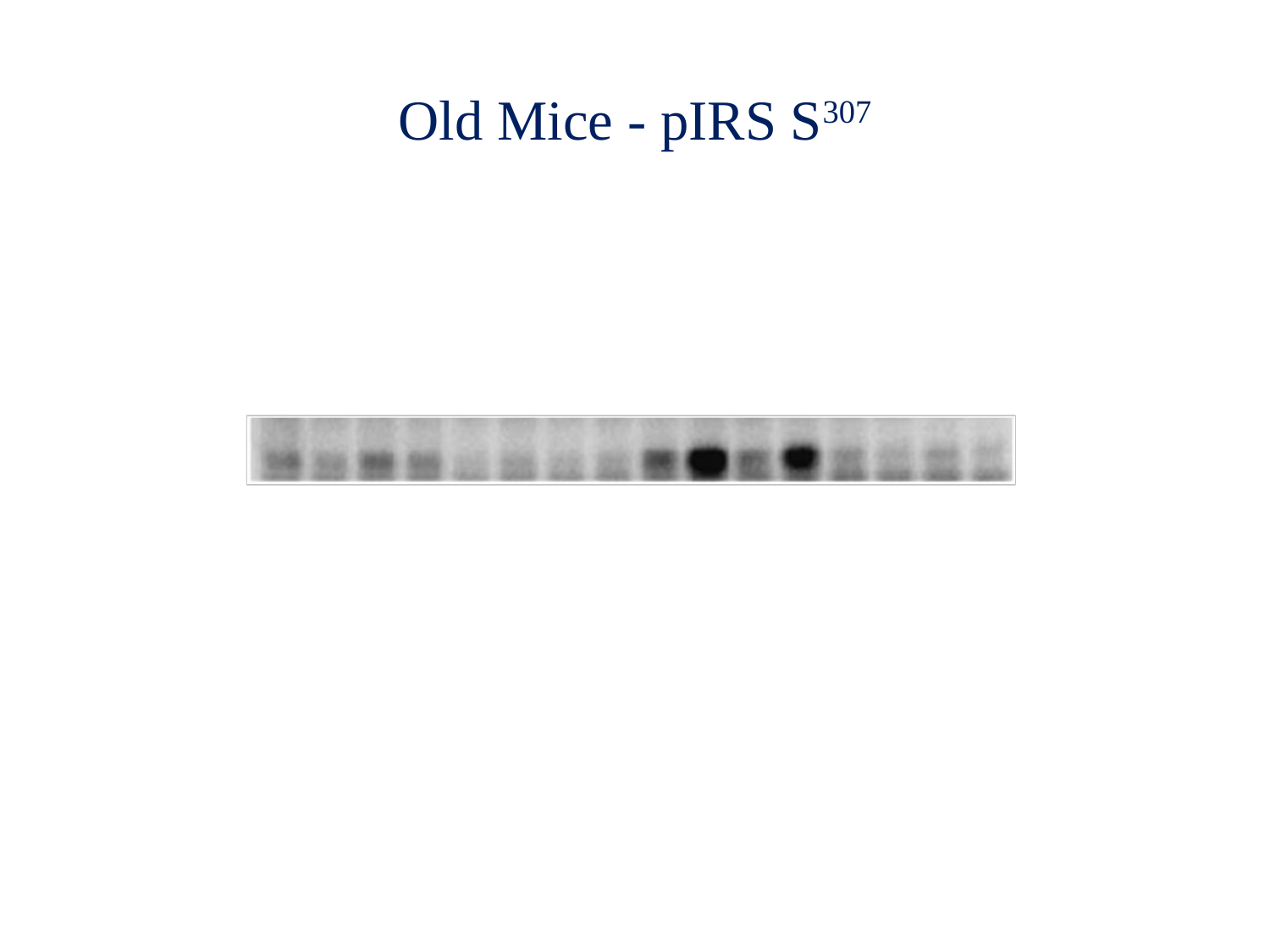

# Old Mice - pIRS S307

## Slide 9
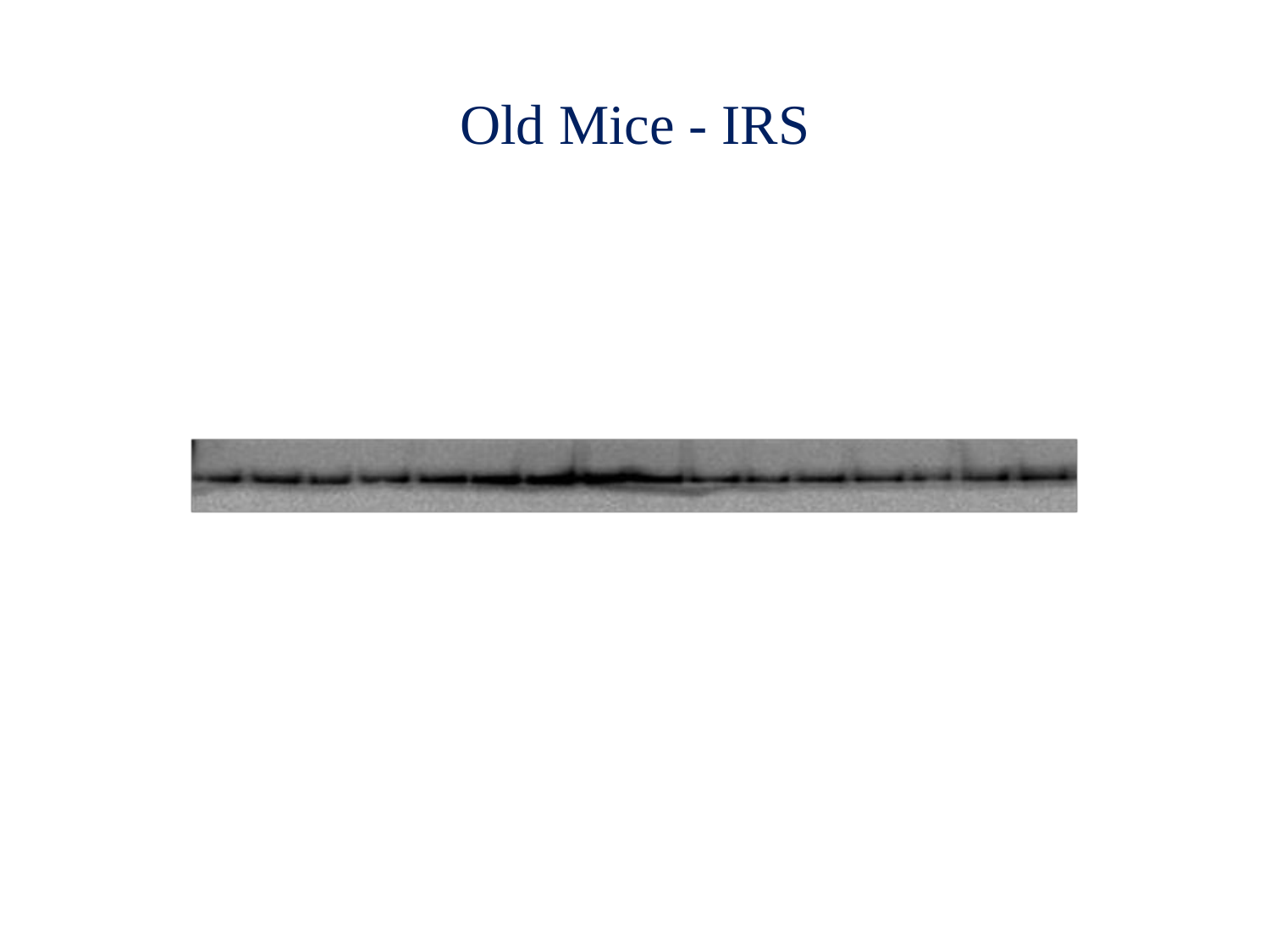

Old Mice - IRS

## Slide 10
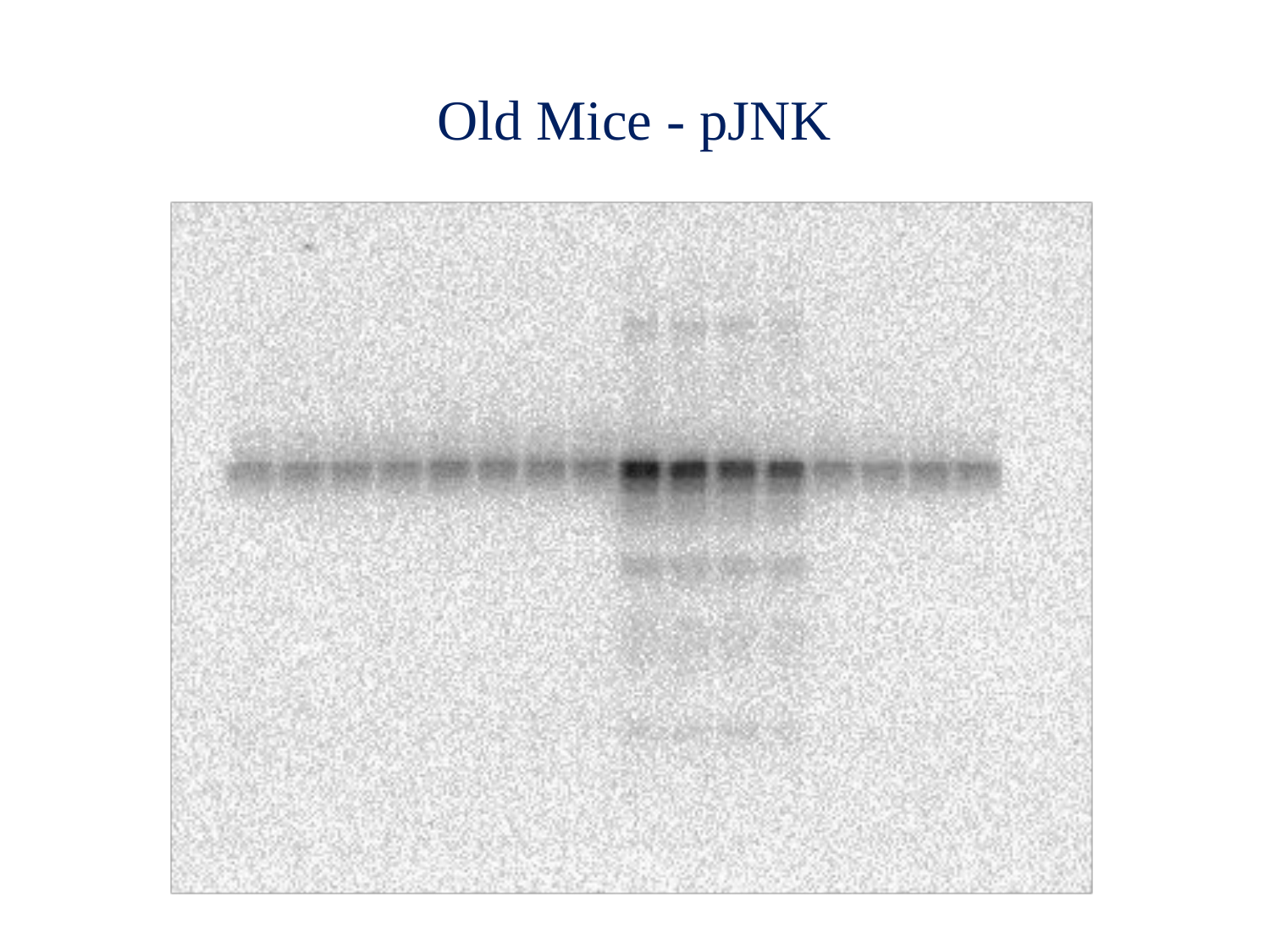

# Old Mice - pJNK

## Slide 11
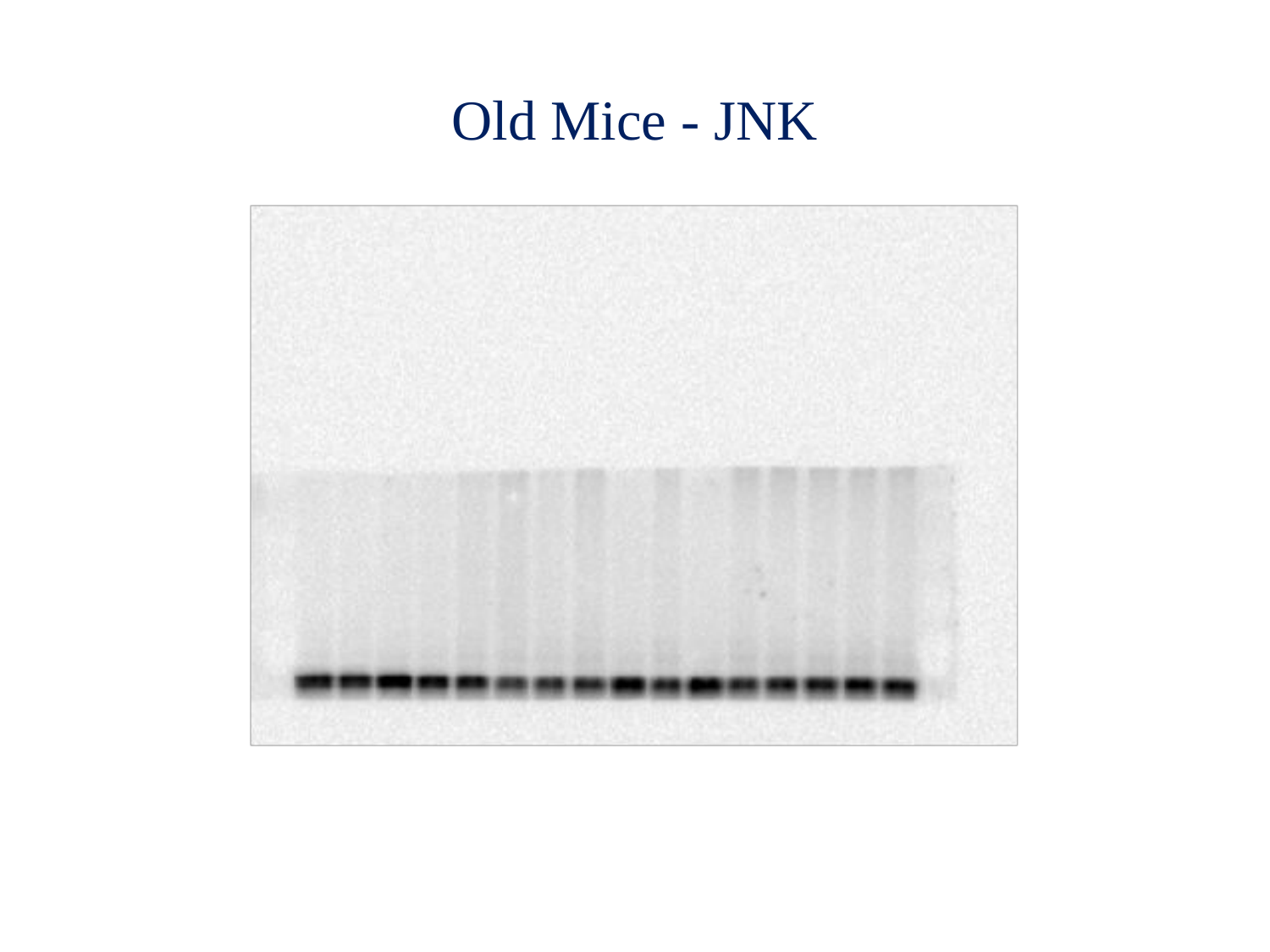

# Old Mice - JNK
